# Emission Characteristics of Air Pollutants and CO_2_ from 11 Cities with Different Economic Development around the Bohai Sea in China from 2008–2017

**DOI:** 10.3390/toxics10090547

**Published:** 2022-09-19

**Authors:** Zongshan Zhao, Qingyang Liu, Jing Lan, Yaru Li

**Affiliations:** 1 College of Environmental Science and Engineering, Qingdao University, Qingdao 266000, China; 2 College of Biology and the Environment, Nanjing Forestry University, Nanjing 210037, China

**Keywords:** CO_2_ emission, air pollution, sustainable development, China

## Abstract

Cities around the Bohai Sea are one of the main population cluster areas in China, which are characterized by high levels of sustainability performance and human capital, as well as resource-intensive industries. In this study, levels of economic development metrics and emissions of air pollutants (BC, CO, NH_3_, NO_x_, OC, PM_2.5_, PM_10_, and SO_2_) and CO_2_ across eleven cities around the Bohai Sea from 2008 to 2017 were compared to illustrate the potential relationships between air pollutants/carbon emissions and socioeconomic developments. Meanwhile, the associations between the levels of economic development metrics (GDP per capita), emissions, and energy use per GDP have also been examined. Large differences across these 11 cities presenting different economic development levels and energy consumption characteristics have been observed. Cities with development dependable on the consumption of fossil fuels and the development of resource-intensive industries have emitted large amounts of air pollutants and CO_2_. Furthermore, the emissions and energy use per GDP for all the cities follow environmental Kuznets curves. The comparison results suggested that the developing cities dependable on resource-intensive industries around the Bohai Sea would obtain greater socioeconomic benefits owing to the interregional cooperation policies under top-down socioeconomic development plans and bottom-up technology development, accompanied by reduced emissions of air pollutants and CO_2_.

## 1. Introduction

Sustainable development of society faces steep challenges regarding climate change and atmospheric pollution with several pollutants accounting for both issues [1,2,3]. Climate change resulting from the emission of greenhouse gases (e.g., CO_2_, CH_4_, N_2_O, O_3_) could reduce food production and pose health burdens to residents through extreme heat and weather events (i.e., floods and droughts) in vulnerable areas where countries are located [1,2,4]. Meanwhile, Low- and Middle-Income Countries (LMICs) pursue economic development with high dependence on the consumption of natural sources (e.g., forests, minerals) and fossil fuels (e.g., coal and crude oil) [5]. A study estimated that LMICs countries were projected to consume primary energy (i.e., fossil fuel) as much as double by 2040 from 2016, while the demand for primary energy in high-income countries may decrease by 8% from 2016 to 2040 [5,6]. High-income countries contributed to high fractions (50–70%) of global greenhouse gas emissions in the atmosphere [3]. Since CO_2_ emissions per capita are found to be correlated with socioeconomic development, large amounts of air pollutants (e.g., PM_2.5_, black carbon (BC), SO_2_, NO_x_) and CO_2_ are projected to be emitted with the development of LMICs countries, which could change regional weather patterns and increase health risks due to exposure to air pollution.

Nearly 95% of cities in LMICs did not achieve the air quality guideline of 10 μg m^−3^ for yearly average PM_2.5_ in 2016, whereas half of the cities in high-income countries met the guideline [7,8,9]. Cities experiencing environmental degradation and climate change have limited capacities to manage and adapt to these risks because of the challenges associated with developments [10].

Prior studies have documented the long-term relationships between GDP per capita and CO_2_ emissions per capita in cities of representative countries worldwide [3,11]. This relationship follows the environmental Kuznets curves (EKC), where emissions first increase, then decrease with income growth [12,13]. Liu et al. [3] demonstrated that CO_2_ emissions per capita were highly associated with GDP per capita and the human development index (HDI) in 184 countries worldwide. Awan et al. [11] examined the validity of the EKC hypothesis using CO_2_ per capita and economic developments in five countries, including China, France, the Russian Federation, the UK, and the USA from 1993 to 2017. A study by Ru et al. [14] indicated that long-term associations between income growth and emissions of SO_2_ and CO_2_ were dependent on energy sectors globally using the country-level emission inventory. However, the emission of BC did not follow the EKC pattern. The emissions of CO_2_ from the industrial and residential sectors, as well as the emissions of SO_2_ from the power and industrial sectors, obeyed the EKC curve, while emissions of SO_2_ and CO_2_ from other sectors did not. 

The United Nations 2030 Agenda appeals for the 17 sustainable development goals (SDG) of cities (e.g., no poverty, good health and well-being, affordable and clean energy, economic growth, climate action) in the inclusive, safe, resilient, and sustainable pathway [15,16,17]. Many countries including China pledge sustainable development by incorporating the 2030 Sustainable Development Goal (SDG) agenda into their national development plan [16]. It was estimated that China may have been responsible for ~30% of global CO_2_ emissions in 2019 [18]. The Chinese central government is committed to reducing the CO_2_ emissions per GDP by 13.5% in 2025 relative to that in 2020, as well as 65% in 2030 compared to 2005, to fulfill the responsibility for the support of global sustainability [18]. In addition, northern citizens in China have been choked with severe air pollution with PM_2.5_ higher than 75 μg m^−3^ over the last few decades [9,19,20]. To achieve the 2030 SDG goals, a top-down scheme of macro-level governance for the priority regions accounting for air pollution and CO_2_ in China is essential and should be recognized for effective local governance and regional coherence of national SDG implementation [17]. 

The Bohai Sea is the largest inland sea in northern China, and its continental shelf is a mining area for natural gas, crude oil, and coal [21,22,23]. The littoral zone of the Bohai Sea is an economically developed region with a high population density in China [24,25]. The socioeconomic development of some cities in this region relies on the large consumption of specific energy resources originating from fossil fuels [26]. Large-scale industries including iron and steel smelting are densely distributed in this region [27,28,29]. For instance, Tangshan in Hebei Province along the littoral zone of the Bohai Sea is the largest producer of iron and steel among cities in the world [30]. It produced approximately 80 million tons of iron and steel in 2012, which is almost equal to the national steel production in the United States [31,32]. Therefore, high-intensity emissions of greenhouse gases and air pollutants from the combustion of fossil fuels have been observed in this region [18,33]. Several field studies have documented extreme air pollution and severe haze events over the coastal region of the Bohai Sea where large-scale industries exist [33,34,35].

Aiming to contribute to co-benefit strategies for city-level sustainable development as well as reductions in emissions of air pollution and CO_2_ in the littoral zone of the Bohai Sea, our study is performed by assessing the relationships between emissions of air pollution/CO_2_ and GDP in 11 representative cities with different development levels and capital structures. The metrics of air pollution include BC, CO, NH_3_, NO_x_, OC, PM_2.5_, PM_10_, and SO_2_ from different sectors (industrial, power, residential, agriculture, and transportation), as well as GDP from the primary sector (agriculture), the secondary sector (mining and manufacturing), and tertiary sector from 2008 to 2017, are adopted in the comprehensive analysis. This study can provide policy guidance for the potential of diverse sustainable development pathways in the context of air pollution and climate mitigation in the short term in those respective cities. 

## 2. Methods

### 2.1. Description of Studied Cities

In this study, 11 cities, namely, Beijing, Tianjin, Dalian, Yingkou, Panjin, Jinzhou, Qinhuangdao, Tangshan, Dongying, Weifang, and Yantai, located along the littoral zone of the Bohai Sea were selected to evaluate the impact of greenhouse gas emissions and air pollution on socioeconomic development from 2008 to 2017 (Table 1). As illustrated in Figure S1, four cities, Dalian, Yingkou, Panjin, and Jinzhou, are in Liaoning Province and are distributed in the northeast of the Bohai Sea. The cities of Qinhuangdao and Tangshan are located in the north of the Bohai Sea and are subordinated to Heibei Province. Beijing and Tianjin are municipalities directly under the Chinese government and are situated in the central location of the Bohai Sea. Three cities, Dongying, Weifang, and Yantai, are affiliated with Shandong Province and are located south of the Bohai Sea. 

### 2.2. Emission Dataset

The Multi-resolution Emission Inventory for China (MEIC) provides gridded total emissions and emissions by sector of greenhouse gases (CO_2_) and air pollutants (i.e., BC, CO, NH_3_, NO_x_, organic carbon (OC), PM_2.5_, PM_10_, and SO_2_) at 0.5° × 0.5°over land by combining the bottom-up technical method with the latest emission inventories in China, respectively [36,37]. The emission inventories in the MEIC include unit-based emission inventories for power plants and cement plants, a high-resolution country-level vehicle emission inventory, a residential combustion emission inventory based on national survey data, and an explicit profile-based non-methanevolatile organic compound (NMVOC) speciation framework [38,39]. The data area available from 2008 to 2017 at http://www.meicmodel.org (accessed on 1 November 2021). We selected the MEIC model because it provides sufficiently high spatial–temporal resolution outputs compared with other alternative models [36]. Furthermore, the accuracies of the results from the MEIC model are proven to be consistent with several outputs estimated from other alternative models (e.g., Transport and Chemical Evolution over the Pacific, TRACE-P; Intercontinental Chemical Transport Experiment-Phase, INTEX-B; Model Inter-Comparison Study for Asia, Hemispheric Transport of Air Pollution, HTAP) [40,41,42,43]. 

The monthly total emissions and emissions by sector of greenhouse gas (i.e., CO_2_) and air pollutants (i.e., BC, CO, NH_3_, NO_x_, OC, PM_2.5_, PM_10_, and SO_2_) for the 11 studied cities from 2008 to 2017 were estimated based on the sum of emissions of all the gridded cells (0.5° × 0.5°) over the city. The dataset of greenhouse gas and air pollutants for Jinzhou City during the periods from 2014 to 2017 was absent. The annual emissions of each city are the sum of monthly emissions in one year. It is speculated that city-level estimates are accurate within 20% using gridded emissions, whereas comparisons across cities tend to be more accurate (<10% error) (Table 2).

### 2.3. Socioeconomic Data

Economic predictors include GDP, GDP of the primary sector (agriculture), GDP of the secondary sector (industry), and GDP of the tertiary sector (excluding agriculture and industry) and are summarized from the China City Statistical Yearbook.

Population data from 2008 to 2017 for 11 cities were obtained from the China Statistical Yearbook and are used to estimate the GDP per capita and GDP per capita of the industry sector using Equations (1) and (2), respectively.

GDP per capita = city-level GDP/city-level population(1)

GDP per capita of the industry sector = GDP of the industry sector/city-level population(2)

The energy use for each city in the form of standard coal consumption was collected from the City Statistical Yearbook and were used to calculate the energy use per GDP with Equation (3). In addition, air pollutants and CO_2_ per GDP in each city were estimated using Equations (4) and (5).

Energy use per GDP = city-level energy use/GDP(3)

Air pollutants per GDP = city-level air pollutant/GDP(4)

CO_2_ per GDP = city-level CO_2_/GDP (5)

### 2.4. Statistics Analysis

We assessed the correlation between energy use per GDP and air pollutants (BC, CO, CO_2_, NH_3_, NO_x_, OC, PM_2.5_, PM_10_, and SO_2_) per GDP as well as CO_2_ per GDP using the Pearson correlation. Moreover, the correlation between economic development metrics including GDP, GDP per capita, GDP of the industry sector, and GDP per capita of the industry sector with air pollution as well as CO_2_ emissions were performed with the Pearson correlation. The statistical analysis was conducted using the Statistical Package for the Social Sciences (IBM SPSS Version 20.0, Armonk, NY, United States).

## 3. Results

### 3.1. Monthly Trends in Emissions

The monthly trends in emissions of BC, CO, OC, PM_2.5_ PM_10_, and SO_2_ in the eleven cities from 2008 to 2017 presented clear cycles with greater emissions in cold seasons (November, December, and January) and lower emissions in warm seasons (June, July, and August). In contrast, higher emissions of NH_3_ were observed in warm seasons (June, July, and August) relative to those of cold seasons (November, December, and January) in all the cities from 2008 to 2017 (Figures S2–S12). The emissions of CO_2_ and NO_x_ from 2008 to 2017 had relatively constant levels with no significant monthly trends (Figures S2–S12). 

These cities presented large differences in the orders of magnitude in emissions of air pollutants (i.e., BC, CO, NH_3_, NO_x_, OC, PM_2.5_, PM_10_, and SO_2_) and CO_2_. For BC, the monthly levels were comparable within the same orders of magnitude, with the highest level in Tianjin (0.8–1.8 metric kilotonnes) and the lowest (0.15–0.25 metric kilotonnes) in Panjin (Table 3). The monthly emissions of NH_3_ and OC were within the orders of magnitude across 11 cities, which varied from 0.8–6.0 metric kilotonnes and 0.2–2.8 metric kilotonnes, respectively. The highest monthly emission of CO_2_ was found in Tangshan (12–19 metric million tonnes), followed by Beijing (8.0–16 metric million tonnes), Tianjin (6.0–16 metric million tonnes), Dongying (2.5–6.0 metric million tonnes), Yantai (4.5–5.6 metric million tonnes), Dalian (4.2–5.5 metric million tonnes), Weifang (3.0–5.0 metric million tonnes), Yingkou (1.4–4.2 metric million tonnes), Jingzhou (1.8–3.0 metric million tonnes), Jinzhou(1.8–3.0 metric million tonnes), and Panjin (0.6–1.0 metric million tonnes). For CO, some cities (Beijing, Tianjin, Dalian, Tangshan, Dongying, Weifang, Yantai, Qinhuangdao) had monthly CO emissions in the range of 50–340 metric kilotonnes, which were higher than more than two times those of other cities, including Jinzhou (20–25 metric kilotonnes), Panjing (2.2–3.1 metric kilotonnes), and Yinkou (15–22 metric kilotonnes).

Tangshan had the highest monthly emissions of NO_x_ (20–35 metric million tonnes), PM_2.5_ (6.0–10.0 metric million tonnes), PM_10_ (7.0–11.0 metric million tonnes), and SO_2_ (15.0–30.0 metric million tonnes) than those of other cities. For the other 10 cities, the emissions of NO_x_, PM_2.5_, PM_10_,and SO_2_ were from high to low in the orders of Tianjin, Beijing, Yantai, Weifang, Dalian, Dongying, Jianzhou, Qinhuangdao, and Panjin (Table 3). 

### 3.2. Annual Trends in Emissions

**Figure 1:**
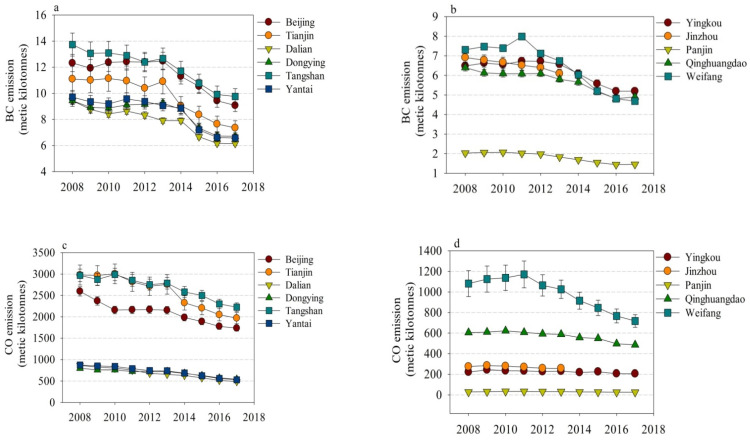

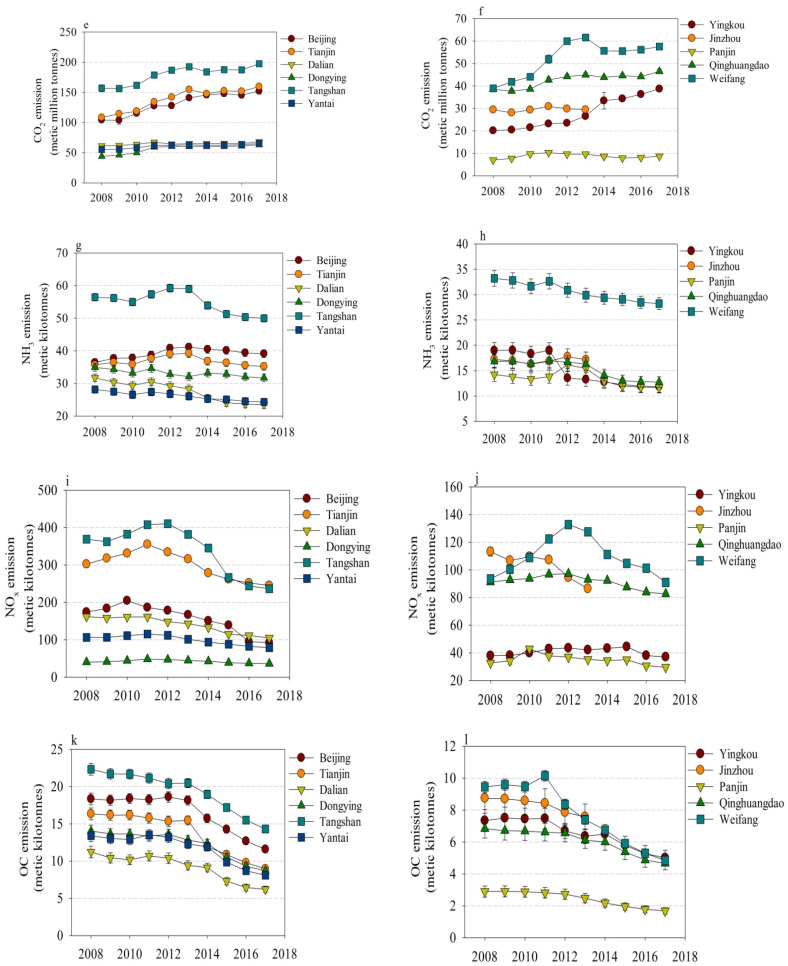

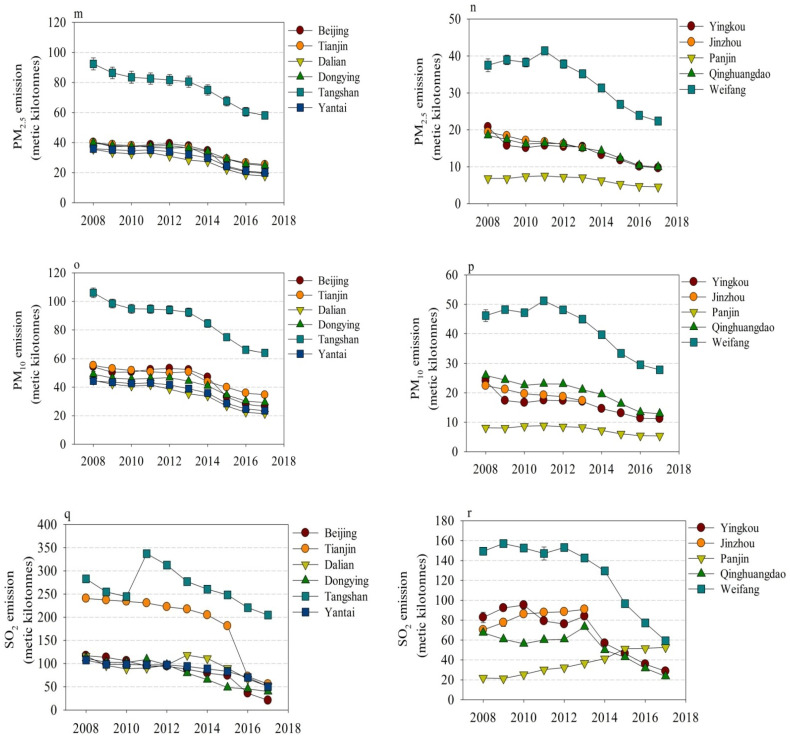
Annual trends in emissions of air pollutants, including BC (**a**,**b**), CO (**c**,**d**), NH_3_ (**g**,**h**), NO_x_ (**i**,**j**), OC (**k**,**l**), PM_2.5_ (**m**,**n**), PM_10_ (**o**,**p**), SO_2_ (**q**,**r**) as well as CO_2_ (**e**,**f**), in the eleven cities during the years of 2018–2017.

The annual levels of air pollutants, including BC, CO, NO_x_, OC, PM_2.5_, and PM_10_,for the eleven cities decreased gradually from 2008 to 2017, though some peak emissions occurred at the periods of 2011 and 2013 for some cities (Figure 1). The annual levels of BC for the 11 cities varied from 2 to 14 metric kilotonnes (Figure 1a,b). The large differences in annual CO emissions were seen across the eleven cities from 2008 to 2017. The levels of annual CO in three cities (Beijing, Tianjin, and Tangshan) ranged from 2000 to 3000 metric kilotonnes, and the levels of annual CO in four cities (Dalian, Dongying, Yantai, and Weifang) were in the range of 200–1500 metric kilotonnes. In comparison, three cities (Panjin, Jinzhou, and Yinkou) emitted an annual CO lower than 200 metric kilotonnes (Figure 1c,d). The levels of NO_x_ in two cities (Tangshan and Tianjin) were higher than 250 metric kilotonnes from 2008 to 2017, and the annual NO_x_ emissions in the range of 80–200 metric kilotonnes were Beijing, Dalian, Yantai, Jinzhou, Qinhuangdao, and Weifang. The annual emissions of NO_x_ in Dongying, Yingkou, and Panjing were in the range of 20–60 metric kilotonnes from 2008 to 2017. Higher annual OC emissions in ten cities (6–25 metric kilotonnes) were seen relative to that (1–3 metric kilotonnes) of Panjin from 2008 to 2017 (Figure 1k,l). Very high annual emissions (60–100 metric kilotonnes) of PM_2.5_ and PM_10_ were observed in Tangshan from 2008 to 2017. The annual emissions of PM_10_ and PM_2.5_ were within the range of 20–60 metric kilotonnes in the other nine cities, while the annual emissions of PM_10_ and PM_2.5_ in Panjin were lower than 10 metric kilotonnes (Figure 1m–p).

The trends in annual emissions of NH_3_ and SO_2_ from 2008 to 2017 showed slight differences across cities. The slight increases in annual emissions of NH_3_ from 35 to 39 metric kilotonnes were seen from 2008 to 2017 in Beijing and Tianjin. The emissions of NH_3_ in Dongying were constant at ~35 metric kilotonnes from 2008 to 2017. The decreasing trend in annual emissions of NH_3_ was seen in Yantai, Dalian, Weifang, Yingkou, Jinzhou, Panjin, and Qinhuangdao from 2008 to 2017. Yet, the annual emission of NH_3_ in Tangshan increased from 55 to 60 metric kilotonnes from 2008 to 2013 and decreased to 50 metric kilotonnes from 2014 to 2017 (Figure 1g,h). With regards to annual SO_2_ emission, the levels in eight cities (Beijing, Dalian, Dongying, Yantai, Jingzhou, Yingkou, Qinhuangdao, and Weifang) decreased steadily from 2008 to 2017. The large variations of annual SO_2_ emissions were seen in Tangshan and Tianjin. Peak annual emissions of SO_2_ were seen at ~350 metric kilotonnes in Tangshan in 2011, while large decreases in annual SO_2_ emissions from 200 to 50 metric kilotonnes were observed in Tianjin from 2015 to 2017. The annual emissions of SO_2_ exhibited an increasing trend from 20 to 40 metric kilotonnes during the periods of 2008 to 2017.

The levels of annual CO_2_ emissions observed were found to increase from 2008 to 2013, while the levels of annual CO_2_ emissions kept at the peak level of 2013 from 2014 to 2017 for all eleven cities. The greatest annual emissions of CO_2_ were seen in Tangshan, followed by Beijing, Tianjin, Dongying, Yantai, Dalian, Weifang, Qinhuangdao, Yingkou, Jinzhou, and Panjin. The largest discrepancies in annual CO_2_ emissions were observed in Tangshan (150–200 metric million tonnes) and Yingkou (5–10 metric million tonnes). 

The contributions of the industry sector accounted for about 20–40% of annual emissions of air pollutants (BC, CO, NH_3_, NO_x_, OC, PM_2.5_, PM_10,_ and SO_2_) as well as CO_2_ for all the eleven cities (Figure S13). The trends in annual emissions of air pollutants (BC, CO, NH_3_, NO_x_, OC, PM_2.5_, PM_10_, and SO_2_), and CO_2_ from 2008 to 2017 were similar to those in total emissions for the eleven cities. Similar trends as those of total annual emissions were also observed in per capita air pollutants and CO_2_, as well as per capita air pollutants and CO_2_ of the industry sector across the eleven cities (Tables S1–S3).

### 3.3. Economic Metrics

Beijing is the capital of China, which had the highest GDP value (1180–2990 billion RMB) during the period of 2008 to 2017 among the eleven cities. Tianjin is a municipality directly under the central government, which has the second-highest GDP value (520–1250 billion RMB) during the period of 2008 to 2017 among the eleven cities. Five cities (Dalian, Tangshan, Dongying, Weifang, and Yantai) had GDP values in the range of 200–700 billion RMB during the period of 2008 to 2017, while four cities (Yingkou, Panjin, Jinzhou, and Qinhuangdao) had the GDP values in the range of 60–150 billion RMB (Table 1). The industry sector contributed about ~20% of the total GDP, which was found to be 300–600 billion RMB during the period of 2008 to 2017. The contributions of the industry sector were responsible for 40–60% of GDP for the other ten cities. Tianjin had the GDP of the industry sector in the range of 300–700 billion RMB, which was higher than those of nine cities, including Dongying (100–300 billion RMB), Yantai (100–300 billion RMB), Dalian (100–300 billion RMB), Tangshan (100–300 billion RMB), Weifang (100–300 billion RMB), Qinhuangdao (50–100 billion RMB), Yingkou (50–100 billion RMB), Jinzhou (50–100 billion RMB), and Panjin (50–100 billion RMB) (Figure S14). The highest GDP per capita during the period of 2008 to 2017 among the eleven cities was Dongying, followed by Beijing, Dalian, Yantai, Tianjin, Panjin, Tangshan, Weifang, Yingkou, Qinhuangdao, and Jinzhou (Table 1). The ranking of GDP of the industry sector per capita for these eleven cities was consistent with that of GDP per capita.

### 3.4. Associations between Economic Metrics and Emissions of Ambient Species

**Figure 2:**
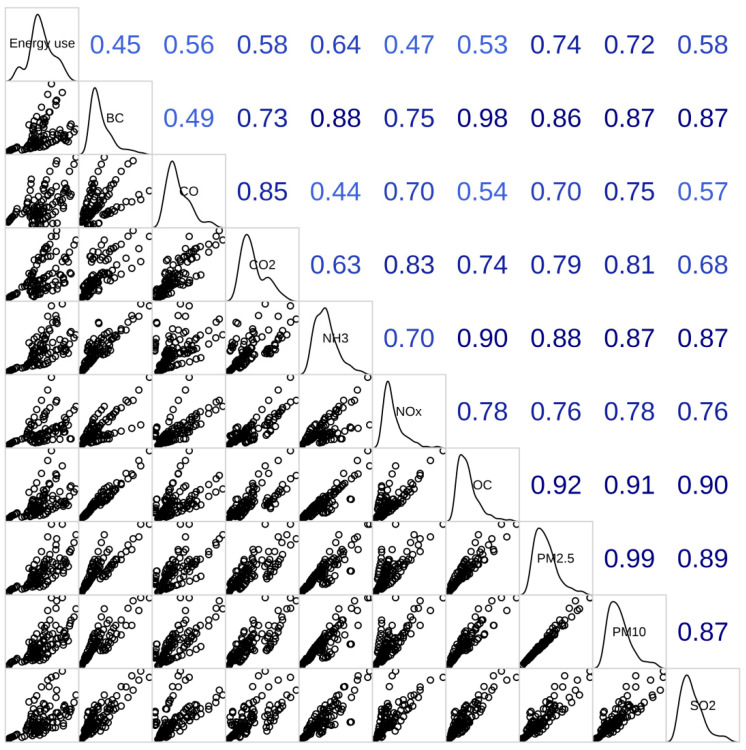
Pearson correlation matrix between energy use per GDP and air pollutants (BC, CO, CO_2_, NH_3_, NO_x_, OC, PM_2.5_, PM_10_ and SO_2_) per GDP, as well as CO_2_ per GDP.

We evaluated the associations between annual economic development metrics including GDP, GDP per capita, GDP of the industry sector, GDP per capita of the industry sector, and annual emissions of ambient species (BC, CO, NH_3_, NO_x_, OC, PM_2.5_, PM_10_, and SO_2_) in 11 cities. GDP was observed to be correlated well with annual emissions of ambient species (BC, CO, NH_3_, NO_x_, OC, PM_2.5_, PM_10_, and SO_2_) (Pearson’s r range, 0.22–0.87, *p* < 0.01). GDP per capita correlated with annual emissions of ambient species (BC, CO, NH_3_, OC, PM_2.5_, PM_10_, and SO_2_) (Pearson’s r range, 0.33–0.51, *p* < 0.05), while GDP per capita was not associated with NO_x_ per capita (*p* > 0.05). Annual emissions of ambient species (BC, CO, NO_x_, OC, PM_2.5_, PM_10_, and SO_2_) exhibited a correlation with GDP of the industry sector, as well as GDP per capita of the industry sector (Pearson’s r range, 0.22–0.86, *p* < 0.05) (Table S4). As shown in Figure 2, the energy use per GDP was significantly associated with air pollutants (BC, CO, CO_2_, NH_3_, NO_x_, OC, PM_2.5_, PM_10_ and SO_2_) per GDP, as well as CO_2_ per GDP with the Pearson r in the range of 0.45–0.90.

### 3.5. Energy Use and Emission per GDP for Cities with Different Development Levels

**Figure 3:**
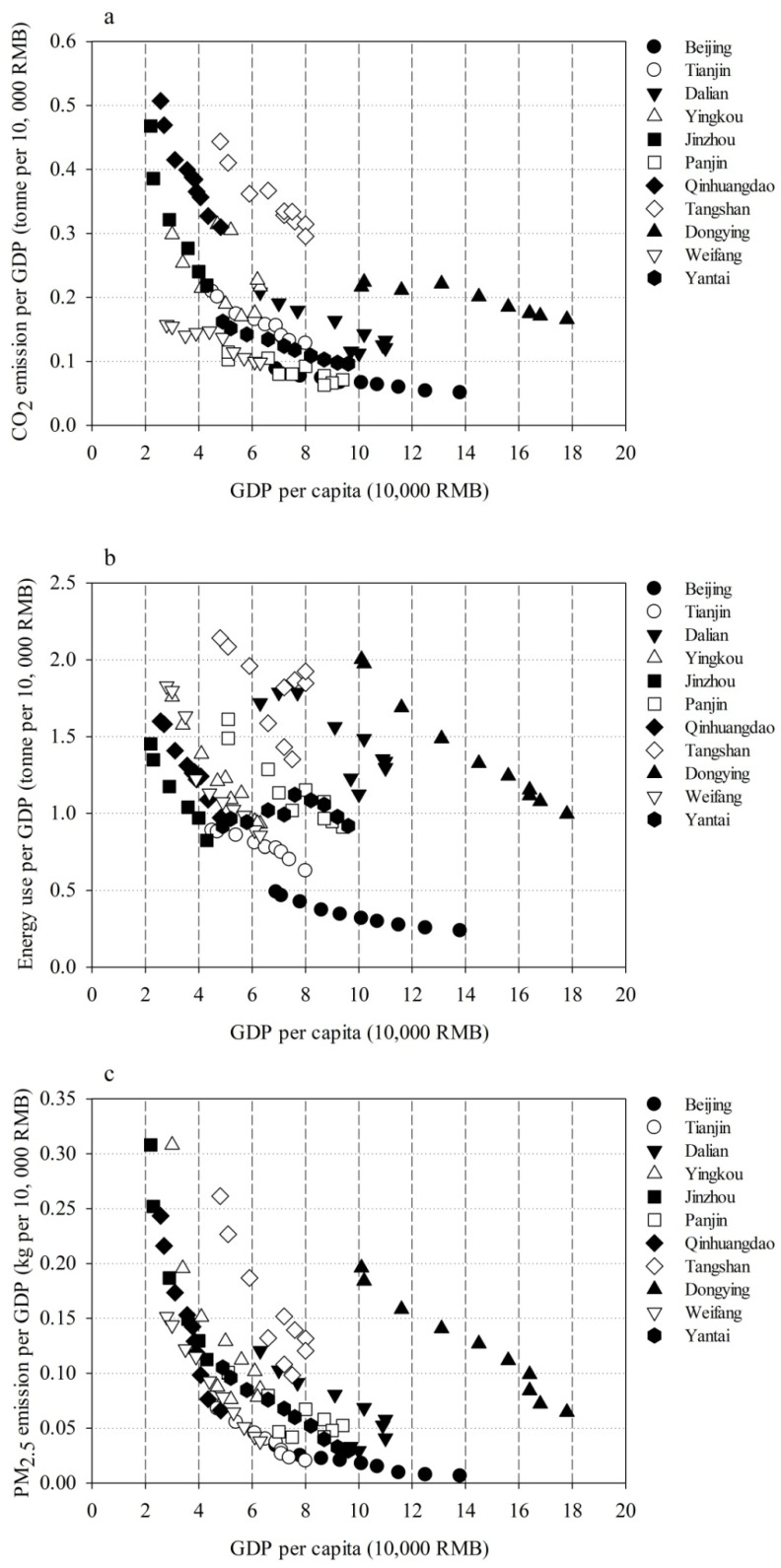
(**a**) CO_2_ per GDP and GDP per capita in the eleven cities from 2008 to 2017. (**b**) Energy use per GDP and GDP per capita in the eleven cities from 2008 to 2017. (**c**) PM_2.5_ per GDP and GDP per capita in the eleven cities from 2008 to 2017.

As shown in Figure 3, CO_2_ emissions per GDP versus cities with different development levels, the energy use per GDP versus cities with different development levels, and PM_2.5_ emissions per GDP versus cities with different development levels are presented. Since the three metrics, including CO_2_ emissions per GDP, energy use per GDP, and PM_2.5_ emissions per GDP versus cities, were highly correlated with each other, the trends of the three metrics against cities with different development levels were found to be comparable. For most cities, including Beijing, Tianjin, Dalian, Jinzhou, Panjin, Qinhuangfao, Dongying, Weifang, and Yantai, the emission and energy use per GDP decreased with the enhancements of economic growth, following the environmental Kuznets curves. The emission and energy use per GDP in Tangshan and Dongying also obeyed the environmental Kuznets curves, but levels of emission and energy use per GDP were found to be higher than those of other cities.

## 4. Discussion

### 4.1. Disparities in Emissions and Economic Development across Cities

During the periods from 2008 to 2017, GDP per capita in China increased steadily from ~25,000 RMB (~3700 US dollars) to ~65,000 RMB (~9500 US dollars) with an average annual growth of ~7%, which reached an average level in middle-income countries [44] (Figure S15a). However, large differences in GDP per capita across the studied 11 cities were observed. Eight cities (Beijing, Tianjin, Dalian, Panjin, Tangshan, Jinzhou, Donging, Weifang, and Tianjin) had GDP per capita higher than the national mean level in China, while the values of GDP per capita in three cities (Yingkou, Jinzhou, and Qinhuangdao) were lower than the national mean level in China (Figure S15b). Since emissions of air pollution and CO_2_ per capita were highly associated with GDP per capita at the initial stage of economic development following the EKC hypothesis [13,45], the large different emissions across the 11 cities were ascribed to the regional disparities in economic development. Our result is consistent with several studies that emissions of air pollution and CO_2_ per capita comply with the EKC hypothesis in cities in Asian countries [7,13,14]. The sum of emissions of air pollution and CO_2_ accounted for 10–30% of total emissions in China. Since city-level emissions of air pollution and CO_2_ across the world are rarely limited, the level comparisons in the emissions of air pollution and CO_2_ across cities around the world could provide a comprehensive evaluation of emissions of air pollution and CO_2_ at a city level in the future. In general, CO_2_ emissions per GDP and emissions of air pollutants per GDP in each city decreased by 30–50% from 2008 to 2017, which illustrated the replacement of old technology with advanced technology driven by economic development could lessen the emissions of CO_2_ and air pollutants [3,46,47]. 

These 11 cities belonging to five regions (Beijing, Tianjin, Liaoning Province, Hebei Province, and Shandong Province) can be simply divided into different city types by characteristics of economic developments, industrial structures, as well as emission levels of air pollutants and CO_2_ [26,36]. Cheng et al. [26] classified 210 cities in China into seven groups based on their sustainability performance in 2016. Beijing is characterized by a high human capital structure with sustainable development driven by high quality and quantity of human capital. Tianjin, four cities (Dalian, Yingkou, Jinzhou, and Panjin) in Liaoning Province, and two cities (Weifang and Yantai) in Shandong Province are featured with high produced capital structure and are highly dependable on the development of the industry. Dongying in Shandong Province is an energy-producing city for crude oil (Table 4). The cities of Tangshan and Qinhuangdao were not grouped in Cheng et al. [26] due to the absences of the data. Tangshan pursues economic development based on abundant coal reserves and steel production [32], while Qinhuangdao is a produced capital city, with development driven by the manufacturing industry [48,49]. 

For a human capital city with high values of GDP per capita, Beijing canexpand its dependency on human capital gradually and abandon the economic development of the industry with high emissions of air pollutants and CO_2_. For those cities associated and reliant on the development of produced capital structure, cities can rely on the industry with low emissions of air pollutants/CO_2_ and improve the mitigation technology for their emission reductions. Proper strategies for reducing ambient species, including PM_2.5_, BC, SO_2_, and CO_2_, from specific sectors could alleviate climate change and air pollution, as well as simultaneously improve socioeconomic development [1,50]. There are several co-benefit policies for the reduction of air pollutants (e.g., PM) and CO_2_ when they are from the same sources. Shindell et al. [1] presented 14 measures that could reduce greenhouse gas emissions in the short term with improvements in human health and food security. Annenberg et al. [50] illustrated that a low-carbon development policy could lead to a decrease in mortality associated with exposure to air pollution and events resulting from climate change, with an increase in the gross domestic product (GDP). However, improper mitigation policies for energy use can result in trade-offs between air pollution and greenhouse gas emissions. For example, switching from coal heating to natural gas was found to reduce PM and BC emissions by 30–80% but increased CO_2_ emissions slightly in some cities, according to prior studies [51,52]. While in a study conducted by Wilson and Stafell, displacing coal with natural gas could lead to reducing per-capita annual emissions by 400 kg CO_2_ between 2015 and 2016 in the UK [53]. For those cities with development dependent on fossil fuels, including coal and crude oil, the increased dependence on the development of resource-intensive industries following ordinary development routes previously can lead to low levels of sustainable development performance. Residents should consider the low levels of sustainability performance in resource-exhausted cities and thus abandon their resource dependency gradually. These cities can pave a development pathway for industries with high-value-added products and low emissions of air pollutants and CO_2_. The shift to low-carbon pathways for resource-dependent cities can be achieved by increasing the capacities to attract high quality and quantity of human capital [53,54,55,56,57].

### 4.2. Mitigation of Air Pollution and CO_2_ Emission at a City Level

For the cities (Yingkou, Jinzhou, and Qinhuangdao) with GDP per capita lower than the national mean level in China, the priorities for the citizens are to seek socioeconomic development in low-carbon development pathways. For the cities (Tangshan and Dongying) with energy use and emission per GDP higher than those other cities, the priorities for the citizens are to pave the low-carbon development with developed technologies. Recent studies indicate that interregional cooperation policies could aid the city to move forward in an economically optimal pathway for carbon emissions reductions and socioeconomic advancement [58,59,60,61]. The pathway for moving to low-carbon development with developed technologies is expected to prioritize PM and GHG reductions, as well as share greater co-benefit in protecting public health. Driscoll et al. [46] found that annual estimated CO_2_ and PM_2.5_ can be reduced by 10–40% in the USA after implementing conventional energy with after-treatment technology and renewable energy in 2417 power plants, avoiding 21–33% of premature deaths associated with air pollution. A study conducted in China suggested that the reductions of 1469 million tonnes of CO_2_ and decreases in 15–22% of all-cause mortality associated with air pollution could be achieved in China around 2030 if low-carbon policies on conventional energy with advanced after-treatment technology were performed [47]. Key steps toward the targets for carbon emissions reductions and socioeconomic advancement in the cities with low levels of development can be supported by the city’s appropriate mitigation action under 14th China’s Five-Year Plans. These development plans can aid the citizens to expand their industrial productivity and promote economic development locally [62].

## 5. Conclusions

Our study presented the differences in emissions of air pollutants and CO_2_ across 11 cities around the Bohai Sea. Beijing, presenting high human capital structures, has emitted total amounts of CO_2_ in the range of 100–150 metric million tonnes and air pollutants (BC, CO, NH_3_, NO_x_, OC, PM_2.5_, PM_10_, and SO_2_) in the range of 1500–2500 metric kilotonnes during the period of 2008–2017. Another eightcities, including Tianjin, Dalian, Panjin, Tangshan, Jinzhou, Donging, Weifang, and Tianjin, characterized by high produced capital structures have produced total amounts of CO_2_ in the range of 40–200 metric million tonnes and air pollutants (BC, CO, NH_3_, NO_x_, OC, PM_2.5_, PM_10_, and SO_2_) in the range of 500–3000 metric kilotonnes during the period of 2008–2017. The three cities (Panjin, Jinzhou, and Qinhuangdao) with GDP per capita lower than the national mean level in China discharged total amounts of CO_2_ in the range of 20–50 metric million tonnes and air pollutants (BC, CO, NH_3_, NO_x_, OC, PM_2.5_, PM_10_, and SO_2_) in the range of 200–500 metric kilotonnes during the period of 2008–2017. The high energy use cities of Tangshan and Dongying corresponded to higher emissions of air pollutants and CO_2_ per GDP than other cities. Cities with high air pollutants and CO_2_ per GDP should pursue short-term policies to reduce air pollution and increase human development. Short-term policies should focus on technological development in carbon reductions and socioeconomic development simultaneously. Three Cities (Yingkou, Jinzhou, and Qinhuangdao) with lower GDP per capita lower than the national mean level in China are essential for promoting the development of cities in low-carbon pathways.

## Figures and Tables

**Table 1:** Description of the socioeconomic status of the eleven cities.

City	Province	Area (km^2^)	Population ^a,b^ (10,000 Persons)	GDP ^a,b^ (Chinese Currency, Billion RMB)	GDP per Capita ^a,b^ (Chinese Currency, 10,000 RMB)
Beijing	/	16,410	1770–2200	1180–2990	6.9–13.8
Tianjin	/	11,966	1170–1440	520–1250	4.5–8.0
Dalian	Liaoning	12,574	613–700	295–605	6.3–10.0
Yingkou	Liaoning	5427	233–243	67–127	3.0–5.4
Panjin	Liaoning	4102	139–143	67–125	5.1–7.6
Jinzhou	Liaoning	10,301	312–305	63–107	2.2–3.5
Qinhuangdao	Hebei	7802	295–311	76–130	2.7–4.8
Tanshan	Hebei	13,472	746–789	354–592	4.8–7.5
Dongying	Shandong	8243	200–215	202–381	10.0–17.8
Weifang	Shandong	16,167	890–936	248–585	2.8–6.2
Yantai	Shandong	13,864	701–706	341–676	4.0–9.5

^a^ 2008–2017. ^b^ The data areobtained from National Bureau of Statistics of China.

**Table 2:** Summary of air pollutants and CO_2_ data used in this study.

Item	Period	Unit	Data Source	Brief Description
BC	2008–2017	Metric tonnes	Multi-resolution Emission Inventory for China (MEIC)	MEIC model generates a database of air pollutants and CO_2_ over China with regular updates using the bottom-up technical method based on a series of improved emission inventory models, which includes unit-based emission inventories for power plants and cement plants, a high-resolution county-level vehicle emission inventory, a residential combustion emission inventory based on national-wide survey data, and an explicit profile-based non-methanevolatile organic compound (NMVOC) speciationframework. The data areavailable at http://www.meicmodel.org (accessed on 1 November 2021).
CO				
CO_2_				
NH_3_				
NO_x_				
OC				
PM_2.5_				
PM_10_				
SO_2_				

**Table 3:** The summary of the monthly concentration of air pollutants (metric kilotonnes) and CO_2_ (metric million tonnes).

City	BC	CO	CO_2_	NH_3_	NO_x_	OC	PM_2.5_	PM_10_	SO_2_
Beijing	0.6–1.5	148–260	8.0–16	2.0–6.0	5.0–18	1.0–2.8	1.8–4.2	2.0–5.6	2.0–12.3
Tianjin	0.8–1.8	80–200	6.0–16	2.0–5.0	22–32	0.8–2.6	1.2–2.2	3.0–5.8	5.0–22.0
Dalian	0.8–1.0	80–100	4.2–5.5	1.8–4.0	11–14	0.5–2.0	2.0–3.2	2.4–4.0	4.8–11.6
Yingkou	0.5–0.7	15–22	1.4–4.2	1.0–2.4	2.4–4.8	0.5–1.1	0.8–1.8	1.0–2.2	3.0–8.2
Panjin	0.15–0.25	2.2–3.1	0.6–1.0	0.8–2.0	2.0–3.2	0.2–0.48	0.4–0.7	0.5–0.9	1.5–7.6
Jinzhou	0.5–0.7	20–25	1.8–3.0	1.2–2.2	6.4–10	0.5–1.4	1.5–1.8	1.6–2.0	2.5–14.0
Qinghuangdao	0.5–0.6	50–65	2.4–4.0	0.8–2.0	3.0–8.0	0.5–0.9	1.0–1.5	1.5–2.2	2.0–7.0
Tanshan	0.8–1.6	200–340	12–19	3.0–7.0	20–35	1.0–3.8	6.0–10.0	7.0–11.0	15.0–30.0
Dongying	0.7–1.0	60–80	2.5–6.0	1.8–4.2	2.8–4.8	0.6–2.2	1.6–4.8	2.0–6.0	3.0–12.0
Weifang	0.4–1.0	150–220	3.0–5.0	1.5–4.0	5.0–11	0.4–1.6	2.0–5.4	3.0–6.0	5.0–15.0
Yantai	0.6–1.1	60–120	4.0–5.6	1.2–3.8	9.0–11	0.6–2.0	1.8–3.8	2.0–4.4	4.0–11.0

**Table 4:** The classification of 11 cities according to the development types.

Types	City ^a^	Reference
Human capital-dominated development	Beijing	[26]
Produced capital-dominated development	Tianjin, Dalian, Yingkou, Jinzhou, and Panjin in Liaoning Province, as well as Weifang and Yantai in Shandong Province	[26]
Energy-producing city	Dongying in Shandong Province	[26]

^a^ The cities of Tangshan and Qinhuangdao were not grouped due to the absences of the data.

## Data Availability

The data and materials could be available at http://www.meicmodel.org (accessed on 1 November 2021) and also be acquired upon the request from the corresponding author.

## Supplementary Materials

The following supporting information can be downloaded at: https://www.mdpi.com/article/10.3390/toxics10090547/s1, Figure S1: The sketch map for the locations of the eleven cities. Figure S2: Monthly trends in emissions of air pollutants including BC by sector (a), CO by sector (b), NH_3_ by sector (d), NO_x_ by sector (e), OC by sector (f), PM_2.5_ by sector (g), PM_10_ by sector (h), SO_2_ by sector (i), as well as CO_2_ by sector (c) in Beijing from the years of 2008–2017. Figure S3: Monthly trends in emissions of air pollutants including BC by sector (a), CO by sector (b), NH_3_ by sector (d), NO_x_ by sector (e), OC by sector (f), PM_2.5_ by sector (g), PM_10_ by sector (h), SO_2_ by sector (i), as well as CO_2_ by sector (c) in Tianjin from the years of 2008–2017. Figure S4: Monthly trends in emissions of air pollutants including BC by sector (a), CO by sector (b), NH_3_ by sector (d), NO_x_ by sector (e), OC by sector (f), PM_2.5_ by sector (g), PM_10_ by sector (h), SO_2_ by sector (i), as well as CO_2_ by sector (c) in Dalian from the years of 2008–2017. Figure S5: Monthly trends in emissions of air pollutants including BC by sector (a), CO by sector (b), NH_3_ by sector (d), NO_x_ by sector (e), OC by sector (f), PM_2.5_ by sector (g), PM_10_ by sector (h), SO_2_ by sector (i), as well as CO_2_ by sector (c) in Yingkou from the years of 2008–2017. Figure S6: Monthly trends in emissions of air pollutants including BC by sector (a), CO by sector (b), NH_3_ by sector (d), NO_x_ by sector (e), OC by sector (f), PM_2.5_ by sector (g), PM_10_ by sector (h), SO_2_ by sector (i), as well as CO_2_ by sector (c) in Panjin from the years of 2008–2017. Figure S7: Monthly trends in emissions of air pollutants including BC by sector (a), CO by sector (b), NH_3_ by sector (d), NO_x_ by sector (e), OC by sector (f), PM_2.5_ by sector (g), PM_10_ by sector (h), SO_2_ by sector (i), as well as CO_2_ by sector (c) in Jinzhou from the years of 2008–2013. Figure S8: Monthly trends in emissions of air pollutants including BC by sector (a), CO by sector (b), NH_3_ by sector (d), NO_x_ by sector (e), OC by sector (f), PM_2.5_ by sector (g), PM_10_ by sector (h), SO_2_ by sector (i), as well as CO_2_ by sector (c) in Qinhuangdao from the years of 2008–2017. Figure S9: Monthly trends in emissions of air pollutants including BC by sector (a), CO by sector (b), NH_3_ by sector (d), NO_x_ by sector (e), OC by sector (f), PM_2.5_ by sector (g), PM_10_ by sector (h), SO_2_ by sector (i), as well as CO_2_ by sector (c) in Tangshan from the years of 2008–2017. Figure S10: Monthly trends in emissions of air pollutants including BC by sector (a), CO by sector (b), NH_3_ by sector (d), NO_x_ by sector (e), OC by sector (f), PM_2.5_ by sector (g), PM_10_ by sector (h), SO_2_ by sector (i), as well as CO_2_ by sector (c) in Dongying from the years of 2008–2017. Figure S11: Monthly trends in emissions of air pollutants including BCby sector (a), COby sector (b), NH_3_ by sector (d), NO_x_ by sector (e), OC by sector (f), PM_2.5_ by sector (g), PM_10_ by sector (h), SO_2_ by sector (i), as well as CO_2_ by sector (c) in Weifang from the years of 2008–2017. Figure S12: Monthly trends in emissions of air pollutants including BC by sector (a), CO by sector (b), NH_3_ by sector (d), NO_x_ by sector (e), OC by sector (f), PM_2.5_ by sector (g), PM_10_ by sector (h), SO_2_ by sector (i), as well as CO_2_ by sector (c) in Yantai from the years of 2008–2017. Figure S13: Contribution of the different sectors to the yearly air pollutants including BC (a), CO (b), NH_3_ (d), NO_x_ (e), OC (f), PM_2.5_ (g), PM_10_ (h), SO_2_ (i), as well as CO_2_ (c) in the eleven cities from the years of 2008–2017. Figure S14: Contributions of the primary sector (agriculture), the secondary sector (industry), and the tertiary sector (excluding agriculture and industry) to GDP from 2008 to 2017 in the11 cities. Figure S15: (a) Changes in GDP per capita from 2008 to 2017 in China. (b) Changes in ratio of GDP per capita and national mean level from 2008 to 2017 in 11 cities. Ratio of GDP per capita and national mean level was estimated using GDP per capita for each city divided GDP per capita in China. Table S1: The summary of the annual level of air pollutants (Metric kilotonnes) of the industry sector and CO_2_ of the industry sector. Table S2: The summary of the annual level of air pollutants (kg per capita) per capita and CO_2_ (tonnes per capita) per capita. Table S3: The summary of the annual concentration of air pollutants (kg per capita) of the industry sector per capita and CO_2_ (tonnes per capita) of the industry sector per capita. Table S4. Correlation (Pearson’s r) between economic development metrics including GDP, GDP per capita, GDP of industry sector, and GDP per capita of industry sector with air pollution, as well as CO_2_ emission.
