# Supplementary material for: Emission Characteristics of Air Pollutants and CO2 from 11 Cities with Different Economic Development around the Bohai Sea in China from 2008–2017
Source: Toxics. 2022 Sep 19;10(9):547. doi: 10.3390/toxics10090547 (PMC9505806; doi:10.3390/toxics10090547)
Supplement: Supplementary file 1 [file toxics-10-00547-s001.zip › toxics-1909161-supplementary.pdf]

toxics

MDPI

Supplementary Materials: Emission Characteristics of Air Pollutants and CO_2 from 11 Cities with Different Economic Development around the Bohai Sea in China from 2008–2017

Zongshan Zhao, Qingyang Liu, Jing Lan and Yaru Li

Table S1. The summary of the annual level of air pollutants (Metric kilotonnes) of the industry sector and CO_2 of the industry sector.

City	BC	CO	CO_2	NH_3	NO_x	OC	$PM_{2.5}$	PM_{10}	SO_2
Beijing	3.8–4.9	940–1336	54–87	/	33–65	1.8–3.8	10–22	16–36	6–39
Tianjin	2.2–3.8	811–1278	52–71	/	83–112	1.3–2.5	11–18	18–30	33–127
Dalian	2.5–4.4	329–644	33–36	/	53–66	2.6–5.9	15–34	40–83	40–85
Yingkou	1.9–3.4	77–119	8–12	/	11–15	1.5–3.1	2.2–5.1	4.8–11.8	15–39
Jinzhou	2.6–3.0	98–116	10–11	/	32–35	2.3–2.6	10–13	13–16	31–39
Panjin	0.4–0.8	6–10	0.8–0.9	/	3.6–4.5	0.3–0.8	1.1–2.9	1.4–3.9	5–20
Qinhuangdao	1.7–2.8	176–232	23–25	/	36–39	1.5–2.5	6–13	8–20	17–47
Tangshan	3.0–5.0	760–1180	71–81	/	79–130	2.4–4.2	28–51	36–69	120–167
Dongying	2.5–4.4	284–445	21–28	/	12–17	2.2–4.1	14–25	18–34	18–67
Weifang	0.9–1.8	226–300	15–24	/	24–42	0.3–0.9	6–13	9–21	39–107
Yantai	2.3–4.6	184–390	30–32	/	36–45	1.8–4.4	10–23	14–31	39–74

Table S2. The summary of the annual level of air pollutants (kg per capita) per capita and CO_2 (tonnes per capita) per capita.

City	BC	CO	CO_2	NH_3	NO_x	OC	$PM_{2.5}$	PM_{10}	SO_2
Beijing	0.4–0.7	80–151	5.7–7.0	1.8–2.1	4.2–10	0.5–1.0	0.8–2.3	1.2–3.1	0.9–6.8
Tianjin	0.4–0.9	126–258	9.4–10	2.2–3.1	15–26	0.5–1.4	1.6–3.4	2.2–4.8	5.2–7.9
Dalian	1.0–2.0	80–183	11–14	3.8–6.7	17–35	1.0–2.3	2.9–7.5	3.5–9.5	8–24
Yingkou	2.1–2.8	84–102	8–15	4.8–8.4	15–18	2.0–3.2	3.9–9.2	4.5–10.7	11–38
Jinzhou	1.9–2.4	82–95	9–10	5.1–6.0	27–39	2.4–3.0	4.8–6.7	5.5–7.8	24–29
Panjin	1.0–1.5	18–24	5–8	8.0–10	20–30	1.1–2.0	3.1–5.1	3.7–6.0	16–36
Qinhuangdao	1.5–2.0	156–204	12–24	4.1–5.6	26–32	1.4–2.3	3.1–6.2	4.1–8.7	7–22
Tangshan	1.2–1.8	282–402	21–25	6.6–7.6	30–53	1.8–3.0	7.3–12	8.1–14	25–38
Dongying	3.1–4.7	255–396	21–29	15–17	16–23	4.0–6.9	11–19	13–24	18–55
Weifang	0.5–0.8	77–124	4.4–6.0	3.0–3.7	10–14	0.5–1.0	2.4–4.2	2.9–5.3	6–17
Yantai	0.9–1.4	75–125	7.9–9.3	3.4–4.0	11–16	1.1–1.9	2.8–5.1	3.3–6.3	7–15

Table S3. The summary of the annual concentration of air pollutants (kg per capita) of the industry sector per capita and CO_2 (tonnes per capita)of the industry sector per capita.

City	BC	CO	CO_2	NH_3	NO_x	OC	$PM_{2.5}$	PM_{10}	SO_2
Beijing	0.1–0.3	30–80	2–4	1–2	2–4	0.2–0.4	0.4–0.8	0.6–1.0	0.4–1.8
Tianjin	0.1–0.4	40–90	4–6	1–2	4–6	0.5–1.4	0.9–1.1	1.1–1.3	2–4
Dalian	0.3–0.6	40–90	4–6	2–3	4–6	0.4–1	0.6–0.9	0.8–1.2	3–5
Yingkou	0.6–1.1	40–80	4–7	2–3	4–6	1–2	0.7–1.1	0.9–1.3	5–8
Jinzhou	0.4–0.8	40–80	4–5	2–4	4–8	1–2	2–5	3–6	5–9
Panjin	0.3–0.8	30–60	3–5	2–5	4–8	0.4–1	2–5	3–6	6–8
Qinhuangdao	0.3–0.5	50–90	5–6	2–4	3–6	1.5–2	2–5	3–6	3–8
Tangshan	0.7–0.9	90–150	5–6	2–5	5–9	2–3	5–8	6–9	6–12
Dongying	0.9–1.3	100–160	5–6	3–6	3–6	2–3	4–7	5–8	7–14
Weifang	0.2–0.4	40–90	3–5	1–2	3–5	0.2–0.4	0.4–0.8	0.5–0.9	3–7
Yantai	0.2–0.5	40–90	5–6	1–2	3–5	0.2–0.4	0.4–0.8	0.5–0.9	3–8

Table S4. Correlation (Pearson's r) between economic development metrics including GDP, GDP per capita, GDP of industry sector, and GDP per capita of industry sector with air pollution, as well as CO_2 emission.

Item	GDP	GDP of the industry sector	Item	GDP per capita	GDP per capita of the industry sector
BC	0.509**	0.448**	BC per capita	0.330**	0.588**
CO	0.566**	0.787**	CO per capita	0.291**	0.602**
CO_2	0.645**	0.860**	CO_2 per capita	0.509**	0.583**
NH_3	0.507**	/	NH_3 per capita	0.509**	/
NO_x	0.337*	0.643**	NO_x per capita	-0.130	0.050**
OC	0.483**	0.216**	OC per capita	0.409**	0.601**
$PM_{2.5}$	0.225*	0.440**	$PM_{2.5}$ per capita	0.424**	0.693**
PM_{10}	0.871**	0.500**	PM_{10} per capita	0.424**	0.664**
SO_2	0.317*	0.441**	SO_2 per capita	0.330*	0.504**

*p < 0.05, ** p < 0.001.

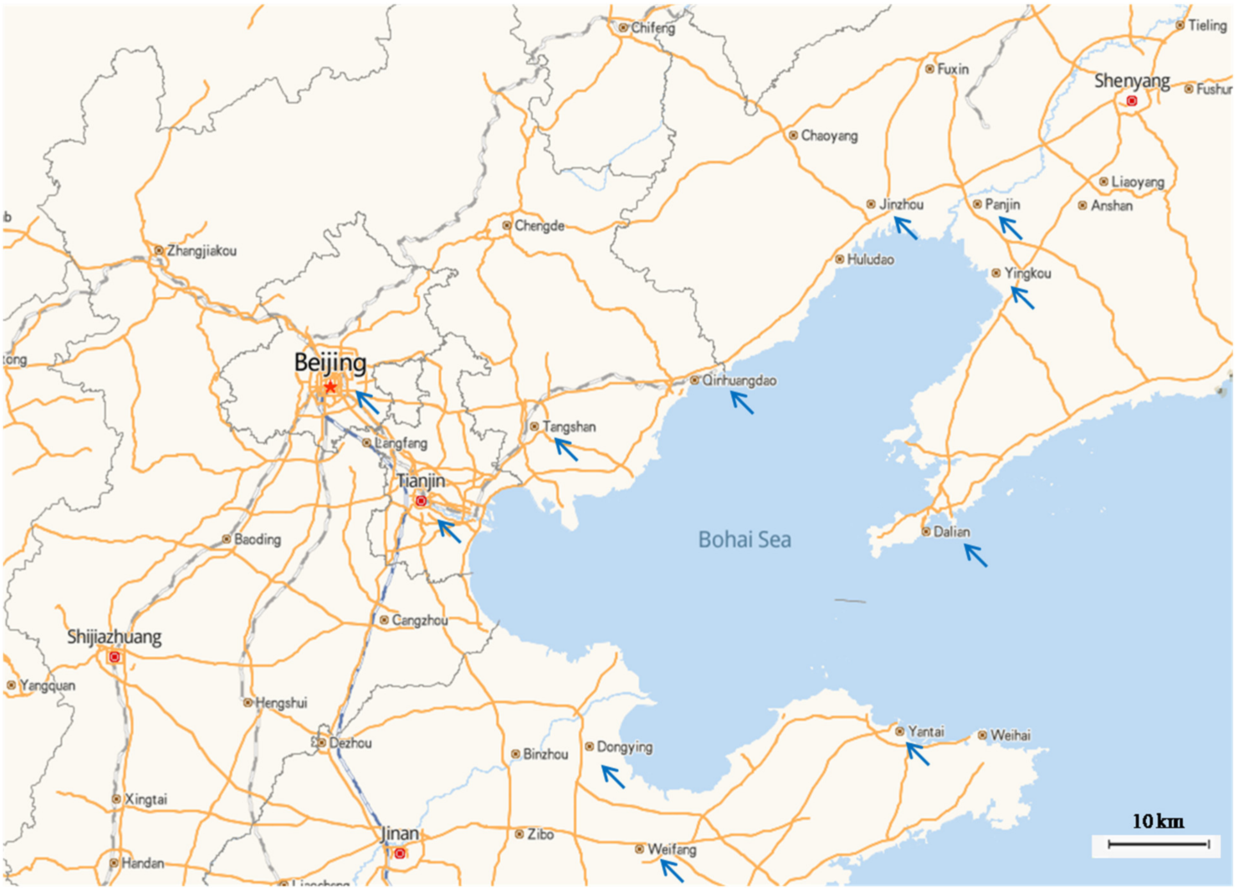

Figure S1. The sketch map for the locations of the eleven cities.

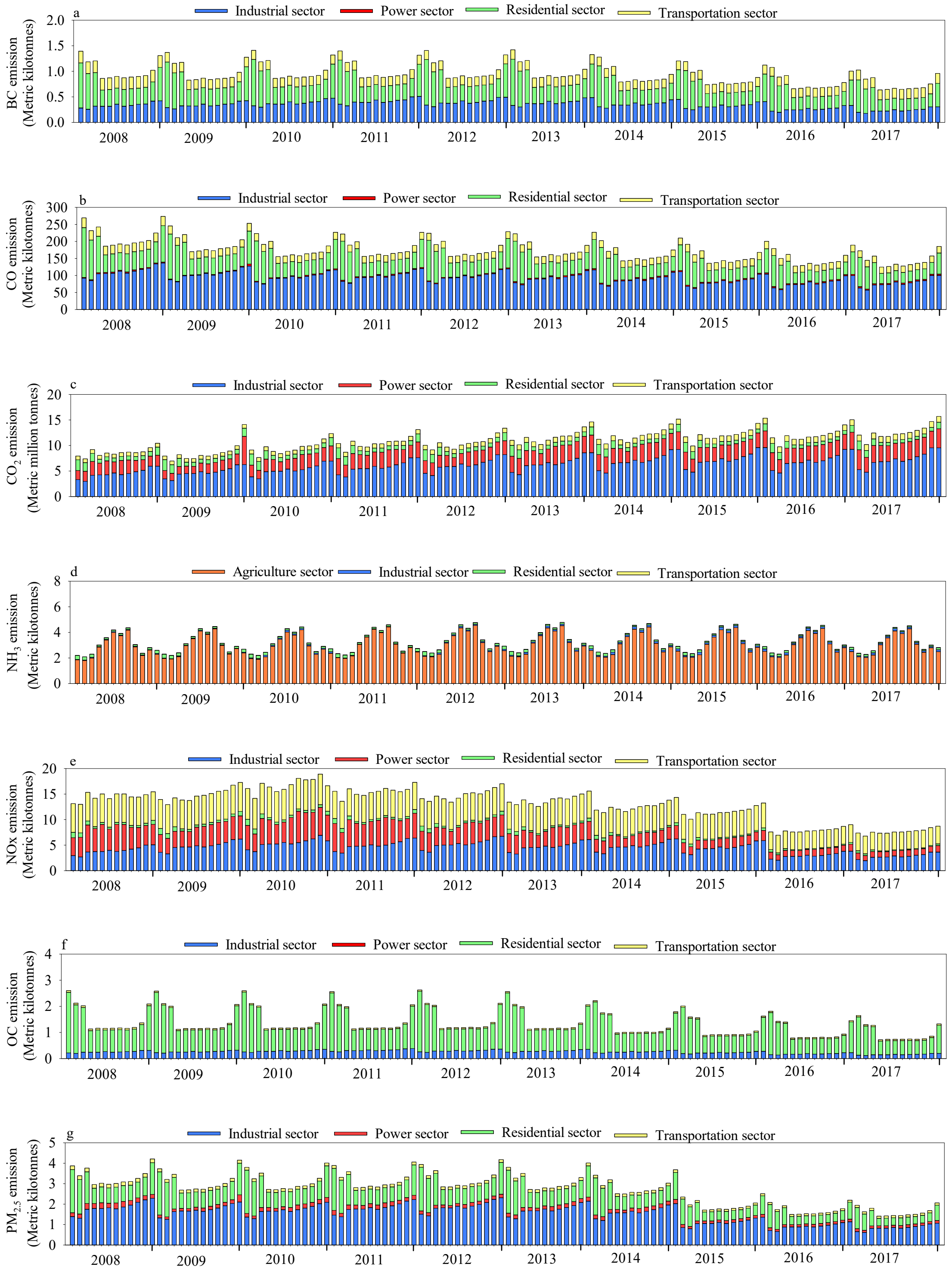
a
Industrial sector
Power sector
Residential sector
Transportation sector
BC emission
(Metric kilotonnes)
b
CO emission
(Metric kilotonnes)
c
CO_2 emission
(Metric million tonnes)
d
Agriculture sector
NH_3 emission
(Metric kilotonnes)
e
NOx emission
(Metric kilotonnes)
f
OC emission
(Metric kilotonnes)
g
$PM_{2.5}$ emission
(Metric kilotonnes)
2008
2009
2010
2011
2012
2013
2014
2015
2016
2017

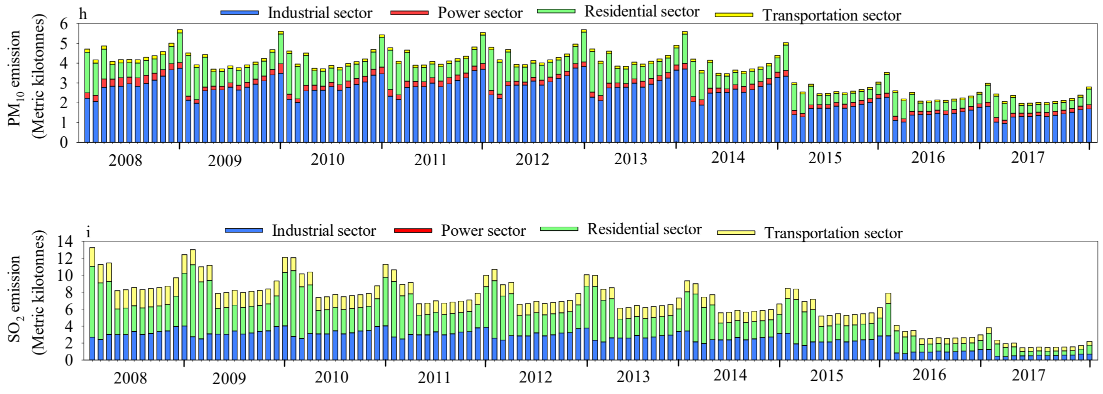

Figure S2. Monthly trends in emissions of air pollutants including BC by sector (a), CO by sector (b), NH_3 by sector (d), NO_X by sector (e), OC by sector (f), $PM_{2.5}$ by sector (g), PM_{10} by sector (h), SO_2 by sector (i), as well as CO_2 by sector (c) in Beijing from the years of 2008-2017.

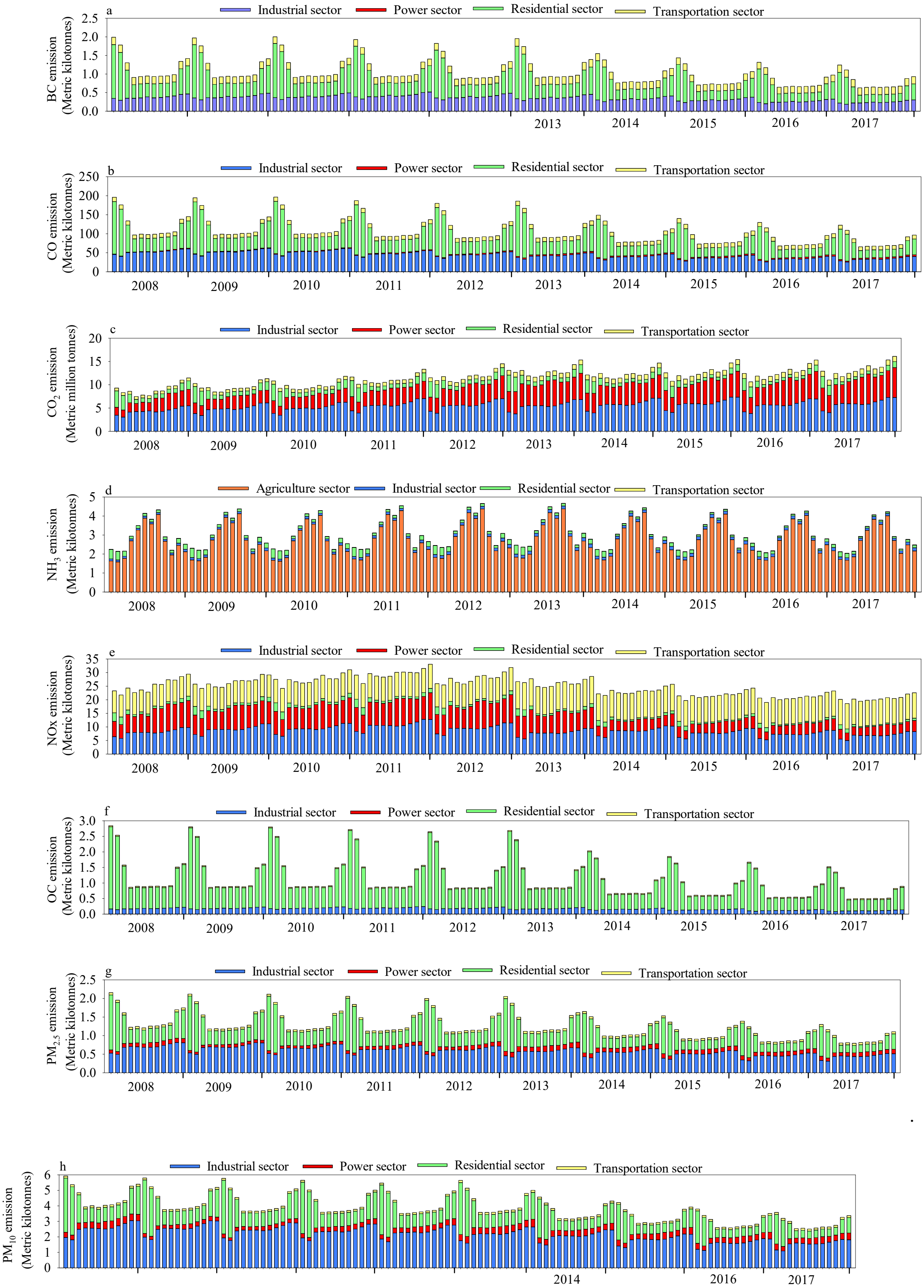
a
Industrial sector
Power sector
Residential sector
Transportation sector
BC emission (Metric kilotonnes)
2013 2014 2015 2016 2017
b
CO emission (Metric kilotonnes)
2008 2009 2010 2011 2012 2013 2014 2015 2016 2017
c
CO2 emission (Metric million tonnes)
d
Agriculture sector
NH3 emission (Metric kilotonnes)
e
NOx emission (Metric kilotonnes)
f
OC emission (Metric kilotonnes)
g
PM2.5 emission (Metric kilotonnes)
h
PM10 emission (Metric kilotonnes)
2014 2016 2017

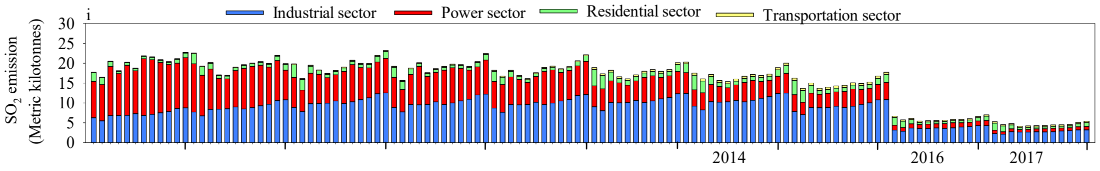

Figure S3. Monthly trends in emissions of air pollutants including BC by sector (a), CO by sector (b), NH_3 by sector (d), NO_X by sector (e), OC by sector (f), $PM_{2.5}$ by sector (g), PM_{10} by sector (h), SO_2 by sector (i), as well as CO_2 by sector (c) in Tianjin from the years of 2008-2017.

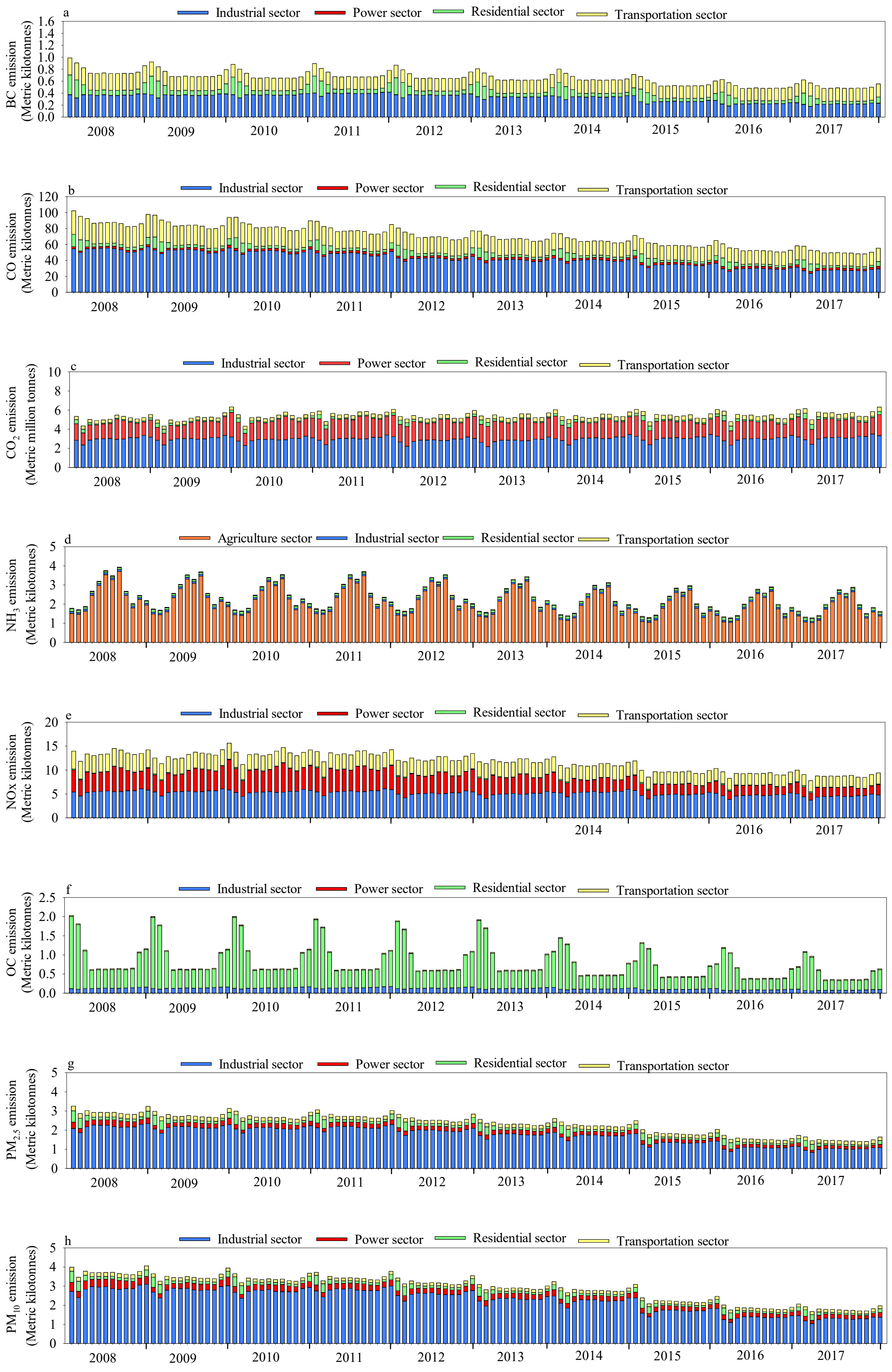
a
Industrial sector
Power sector
Residential sector
Transportation sector
BC emission (Metric kilotonnes)
b
CO emission (Metric kilotonnes)
c
CO_2 emission (Metric million tonnes)
d
Agriculture sector
NH_3 emission (Metric kilotonnes)
e
NOx emission (Metric kilotonnes)
f
OC emission (Metric kilotonnes)
g
$PM_{2.5}$ emission (Metric kilotonnes)
h
PM_{10} emission (Metric kilotonnes)
2008
2009
2010
2011
2012
2013
2014
2015
2016
2017

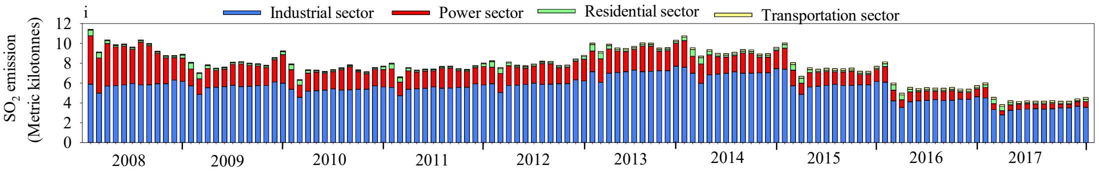

Figure S4. Monthly trends in emissions of air pollutants including BC by sector (a), CO by sector (b), NH_3 by sector (d), NO_X by sector (e), OC by sector (f), $PM_{2.5}$ by sector (g), PM_{10} by sector (h), SO_2 by sector (i), as well as CO_2 by sector (c) in Dalian from the years of 2008-2017.

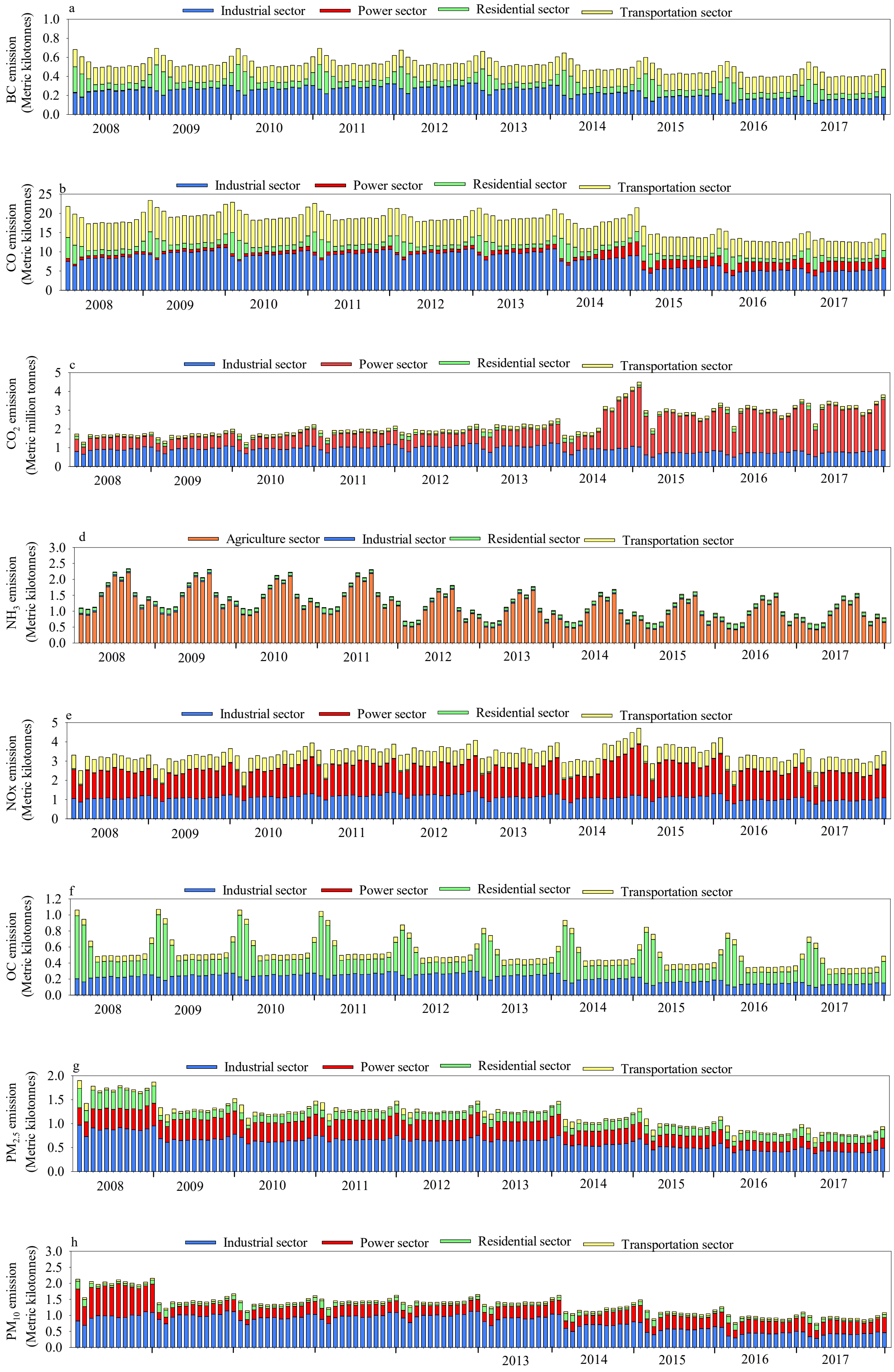
a
Industrial sector
Power sector
Residential sector
Transportation sector
BC emission (Metric kilotonnes)
b
CO emission (Metric kilotonnes)
c
CO2 emission (Metric million tonnes)
d
Agriculture sector
NH3 emission (Metric kilotonnes)
e
NOx emission (Metric kilotonnes)
f
OC emission (Metric kilotonnes)
g
PM2.5 emission (Metric kilotonnes)
h
PM10 emission (Metric kilotonnes)
2008
2009
2010
2011
2012
2013
2014
2015
2016
2017

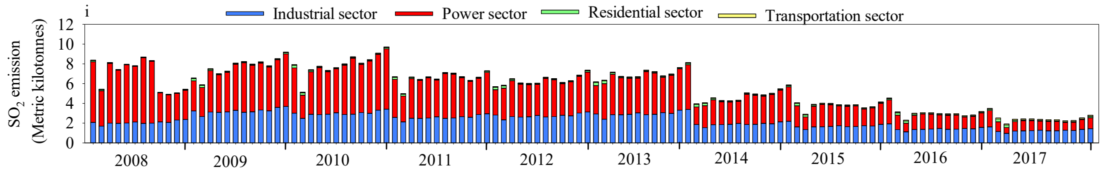

Figure S5. Monthly trends in emissions of air pollutants including BC by sector (a), CO by sector (b), NH_3 by sector (d), NO_X by sector (e), OC by sector (f), $PM_{2.5}$ by sector (g), PM_{10} by sector (h), SO_2 by sector (i), as well as CO_2 by sector (c) in Yingkou from the years of 2008-2017.

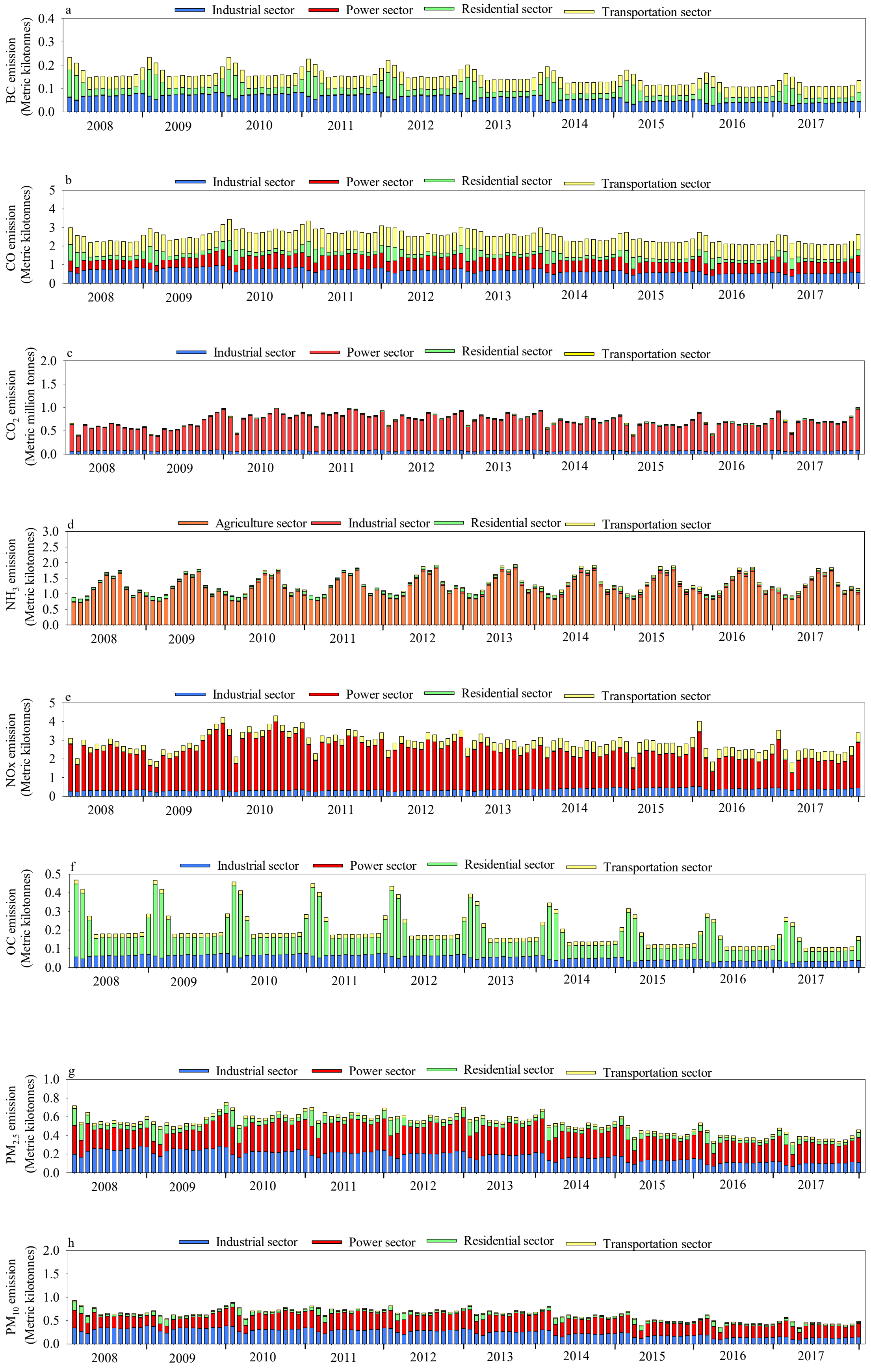
a
Industrial sector
Power sector
Residential sector
Transportation sector
BC emission (Metric kilotonnes)
b
CO emission (Metric kilotonnes)
c
CO_2 emission (Metric million tonnes)
d
Agriculture sector
NH_3 emission (Metric kilotonnes)
e
NOx emission (Metric kilotonnes)
f
OC emission (Metric kilotonnes)
g
$PM_{2.5}$ emission (Metric kilotonnes)
h
PM_{10} emission (Metric kilotonnes)
2008
2009
2010
2011
2012
2013
2014
2015
2016
2017

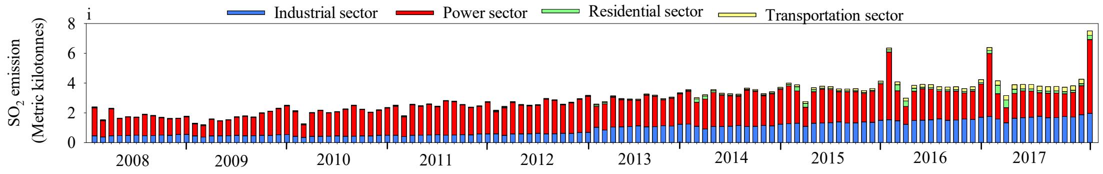

Figure S6. Monthly trends in emissions of air pollutants including BC by sector (a), CO by sector (b), NH_3 by sector (d), NO_X by sector (e), OC by sector (f), $PM_{2.5}$ by sector (g), PM_{10} by sector (h), SO_2 by sector (i), as well as CO_2 by sector (c) in Panjin from the years of 2008-2017.

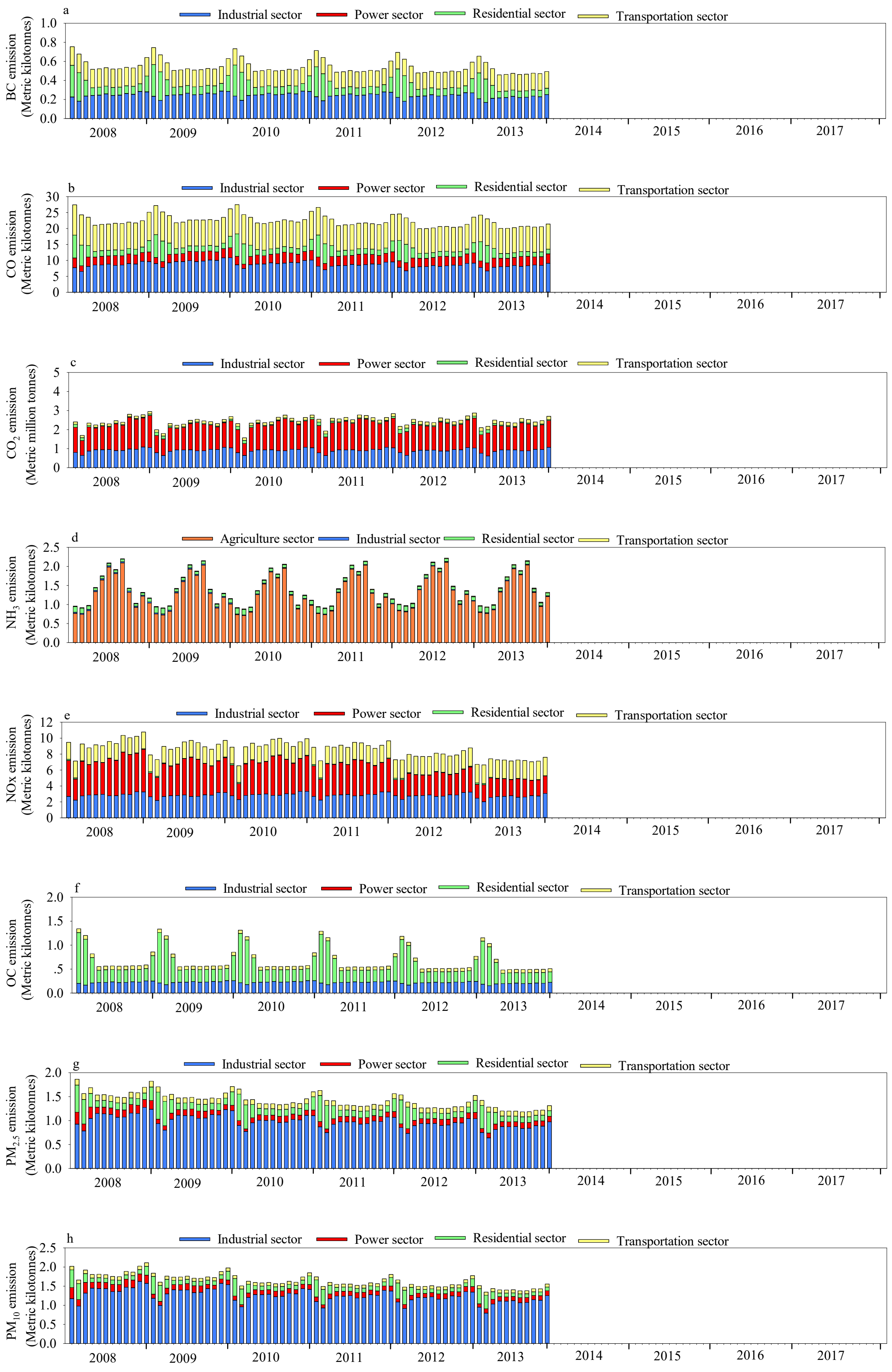
a
Industrial sector
Power sector
Residential sector
Transportation sector
BC emission (Metric kilotonnes)
1.0 0.8 0.6 0.4 0.2 0.0
b
CO emission (Metric kilotonnes)
30 25 20 15 10 5 0
c
CO_2 emission (Metric million tonnes)
5 4 3 2 1 0
d
Agriculture sector
NH_3 emission (Metric kilotonnes)
2.5 2.0 1.5 1.0 0.5 0.0
e
NOx emission (Metric kilotonnes)
12 10 8 6 4 2 0
f
OC emission (Metric kilotonnes)
2.0 1.5 1.0 .5 0.0
g
$PM_{2.5}$ emission (Metric kilotonnes)
2.0 1.5 1.0 0.5 0.0
h
PM_{10} emission (Metric kilotonnes)
2.5 2.0 1.5 1.0 0.5 0.0
2008 2009 2010 2011 2012 2013 2014 2015 2016 2017

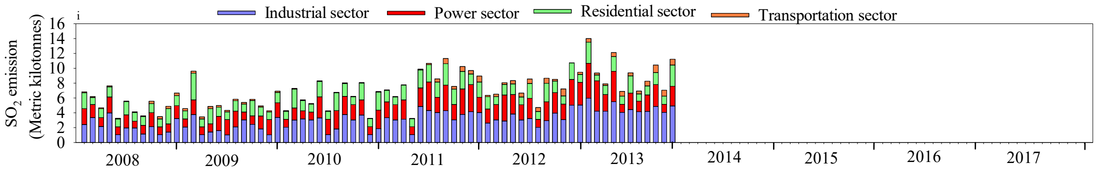

Figure S7. Monthly trends in emissions of air pollutants including BC by sector (a), CO by sector (b), NH_3 by sector (d), NO_X by sector (e), OC by sector (f), $PM_{2.5}$ by sector (g), PM_{10} by sector (h), SO_2 by sector (i), as well as CO_2 by sector (c) in Jinzhou from the years of 2008-2013.

.

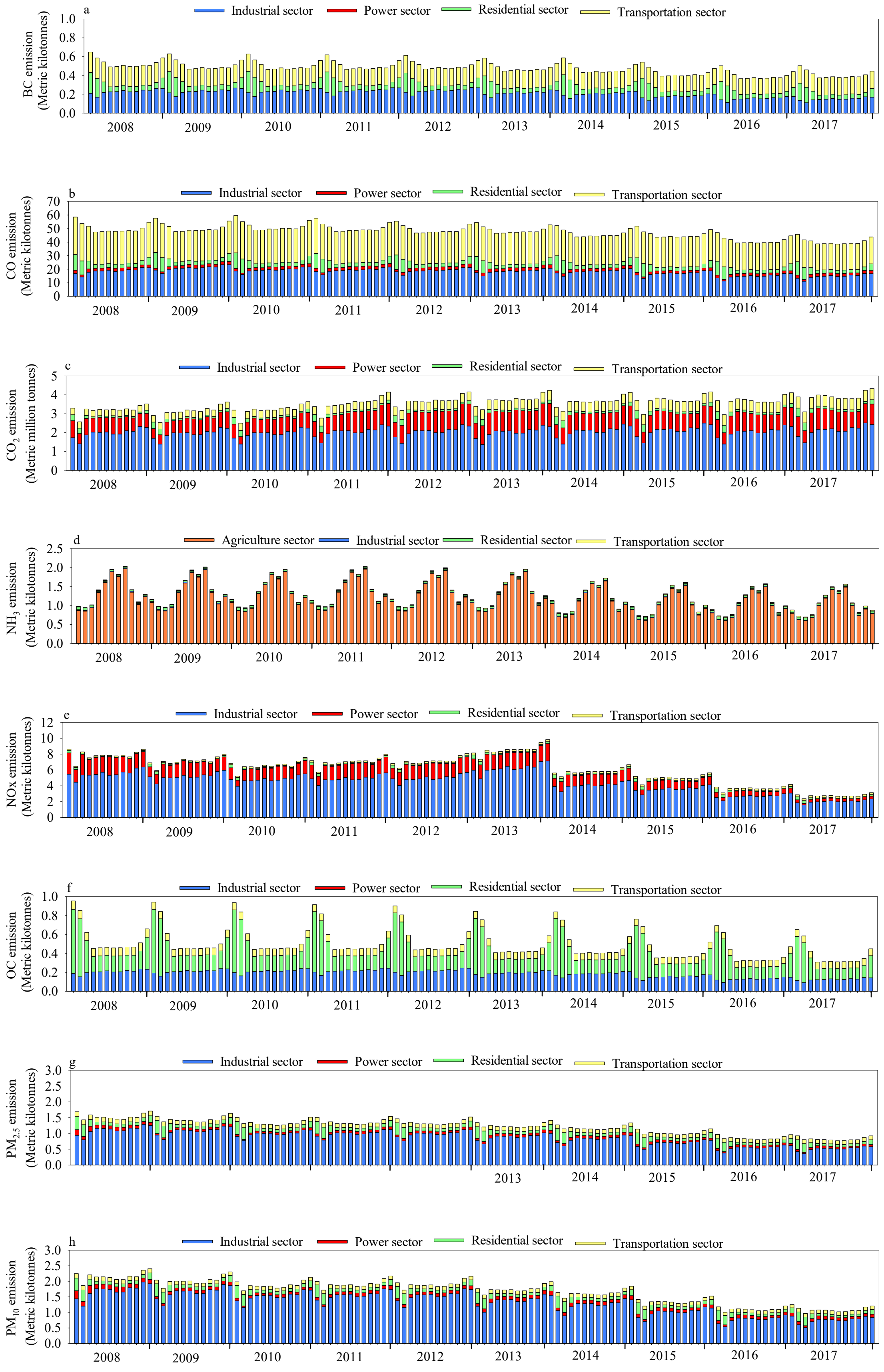
a
Industrial sector
Power sector
Residential sector
Transportation sector
BC emission (Metric kilotonnes)
b
CO emission (Metric kilotonnes)
c
CO_2 emission (Metric million tonnes)
d
Agriculture sector
NH_3 emission (Metric kilotonnes)
e
NOx emission (Metric kilotonnes)
f
OC emission (Metric kilotonnes)
g
$PM_{2.5}$ emission (Metric kilotonnes)
h
PM_{10} emission (Metric kilotonnes)
2008
2009
2010
2011
2012
2013
2014
2015
2016
2017

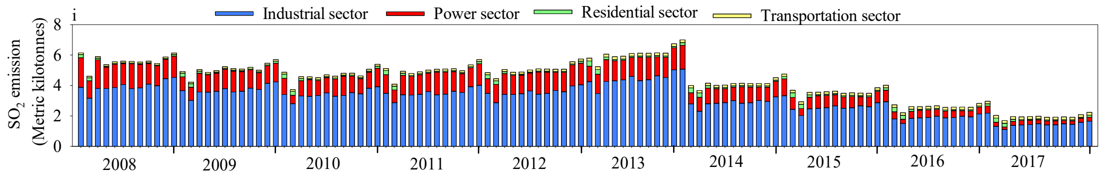

Figure S8. Monthly trends in emissions of air pollutants including BC by sector (a), CO by sector (b), NH_3 by sector (d), NO_X by sector (e), OC by sector (f), $PM_{2.5}$ by sector (g), PM_{10} by sector (h), SO_2 by sector (i), as well as CO_2 by sector (c) in Qinhuangdao from the years of 2008-2017.

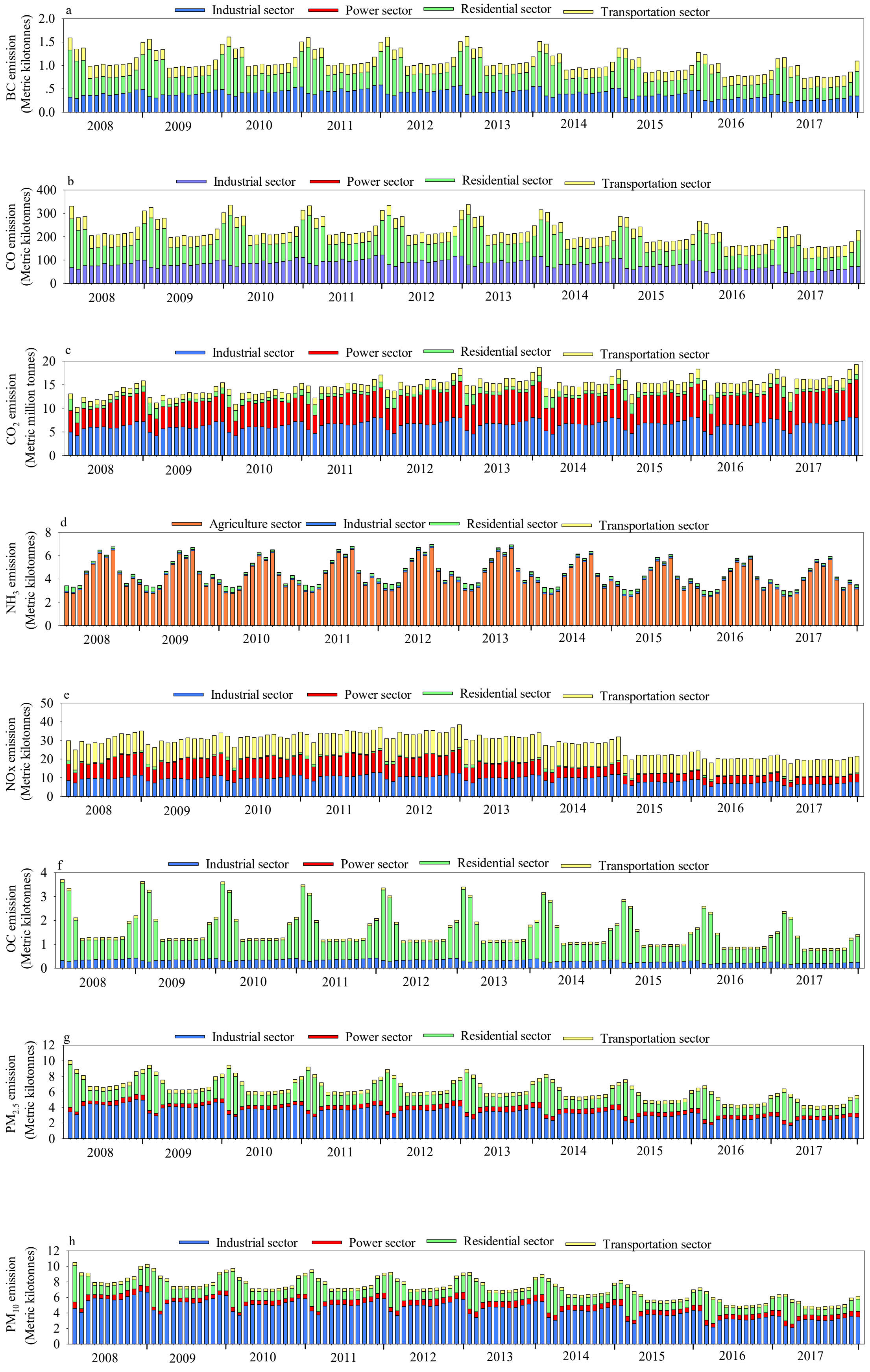
a
Industrial sector
Power sector
Residential sector
Transportation sector
BC emission (Metric kilotonnes)
b
CO emission (Metric kilotonnes)
c
CO_2 emission (Metric million tonnes)
d
Agriculture sector
NH_3 emission (Metric kilotonnes)
e
NOx emission (Metric kilotonnes)
f
OC emission (Metric kilotonnes)
g
$PM_{2.5}$ emission (Metric kilotonnes)
h
PM_{10} emission (Metric kilotonnes)
2008
2009
2010
2011
2012
2013
2014
2015
2016
2017

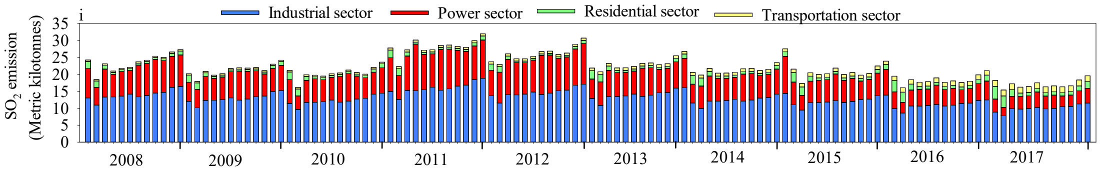

Figure S9. Monthly trends in emissions of air pollutants including BC by sector (a), CO by sector (b), NH_3 by sector (d), NO_X by sector (e), OC by sector (f), $PM_{2.5}$ by sector (g), PM_{10} by sector (h), SO_2 by sector (i), as well as CO_2 by sector (c) in Tangshan from the years of 2008-2017.

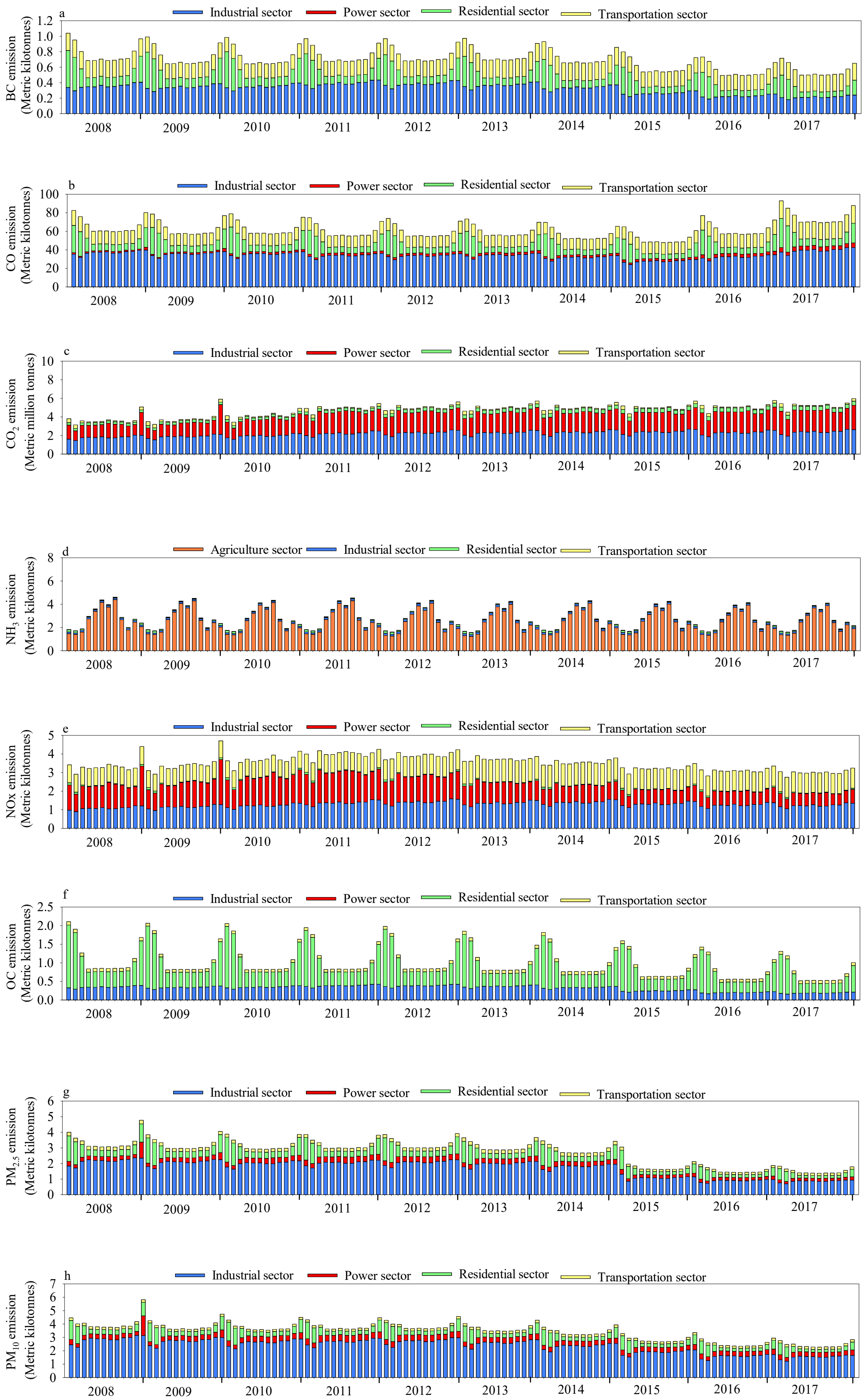
a
Industrial sector
Power sector
Residential sector
Transportation sector
BC emission (Metric kilotonnes)
b
CO emission (Metric kilotonnes)
c
CO_2 emission (Metric million tonnes)
d
Agriculture sector
NH_3 emission (Metric kilotonnes)
e
NOx emission (Metric kilotonnes)
f
OC emission (Metric kilotonnes)
g
$PM_{2.5}$ emission (Metric kilotonnes)
h
PM_{10} emission (Metric kilotonnes)
2008
2009
2010
2011
2012
2013
2014
2015
2016
2017

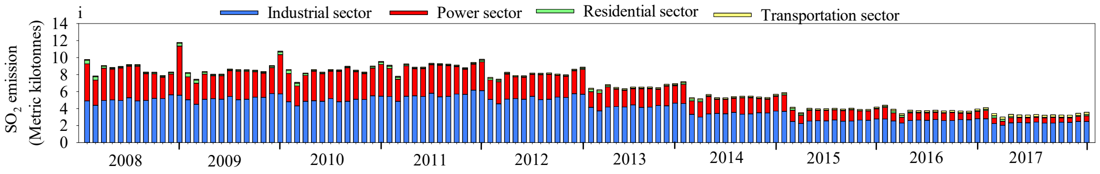

Figure S10. Monthly trends in emissions of air pollutants including BC by sector (a), CO by sector (b), NH_3 by sector (d), NO_X by sector (e), OC by sector (f), $PM_{2.5}$ by sector (g), PM_{10} by sector (h), SO_2 by sector (i), as well as CO_2 by sector (c) in Dongying from the years of 2008-2017.

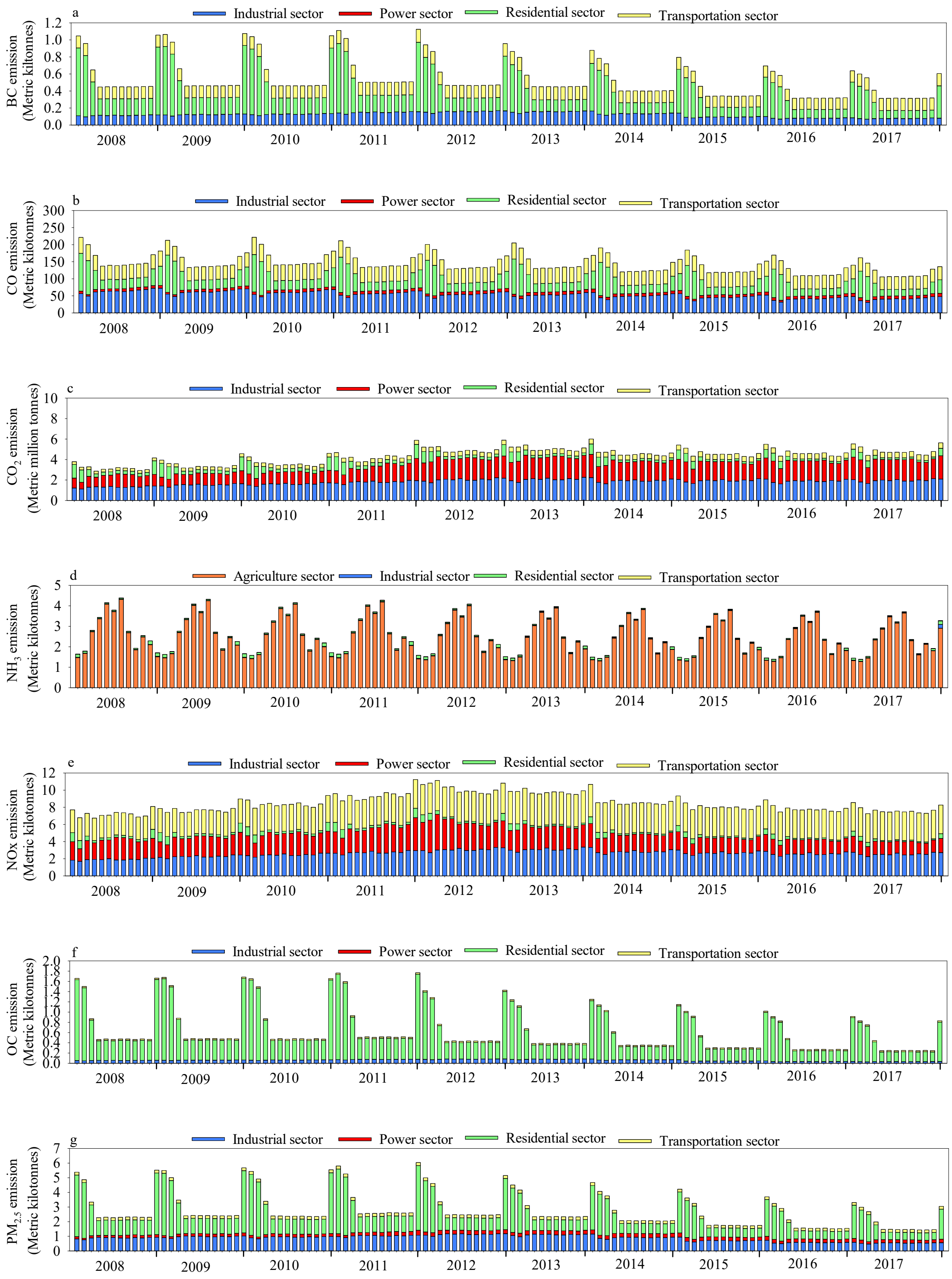
a
Industrial sector
Power sector
Residential sector
Transportation sector
BC emission
(Metric kiltonnes)
1.2
1.0
0.8
0.6
0.4
0.2
0.0
2008
2009
2010
2011
2012
2013
2014
2015
2016
2017
b
Industrial sector
Power sector
Residential sector
Transportation sector
CO emission
(Metric kilotonnes)
300
250
200
150
100
50
0
2008
2009
2010
2011
2012
2013
2014
2015
2016
2017
c
Industrial sector
Power sector
Residential sector
Transportation sector
CO_2 emission
(Metric million tonnes)
10
8
6
4
2
0
2008
2009
2010
2011
2012
2013
2014
2015
2016
2017
d
Agriculture sector
Industrial sector
Residential sector
Transportation sector
NH_3 emission
(Metric kilotonnes)
5
4
3
2
1
0
2008
2009
2010
2011
2012
2013
2014
2015
2016
2017
e
Industrial sector
Power sector
Residential sector
Transportation sector
NOx emission
(Metric kilotonnes)
12
10
8
6
4
2
0
2008
2009
2010
2011
2012
2013
2014
2015
2016
2017
f
Industrial sector
Power sector
Residential sector
Transportation sector
OC emission
(Metric kilotonnes)
2.0
1.8
1.6
1.4
1.2
1.0
0.8
0.6
0.4
0.2
0.0
2008
2009
2010
2011
2012
2013
2014
2015
2016
2017
g
Industrial sector
Power sector
Residential sector
Transportation sector
$PM_{2.5}$ emission
(Metric kilotonnes)
7
6
5
4
3
2
1
0
2008
2009
2010
2011
2012
2013
2014
2015
2016
2017

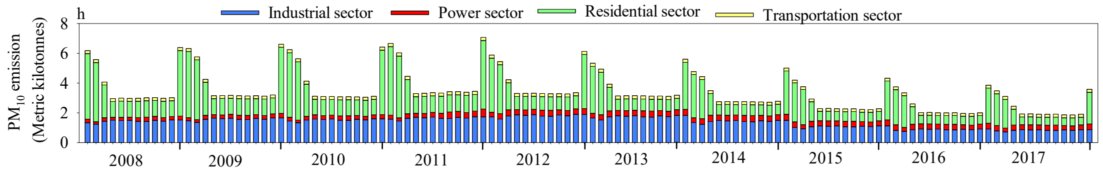

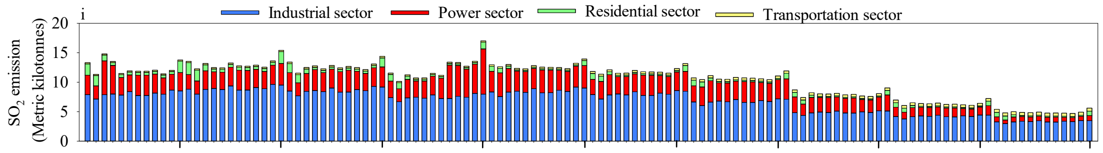

Figure S11. Monthly trends in emissions of air pollutants including BC by sector (a), CO by sector (b), NH_3 by sector (d), NO_X by sector (e), OC by sector (f), $PM_{2.5}$ by sector (g), PM_{10} by sector (h), SO_2 by sector (i), as well as CO_2 by sector (c) in Weifang from the years of 2008-2017.

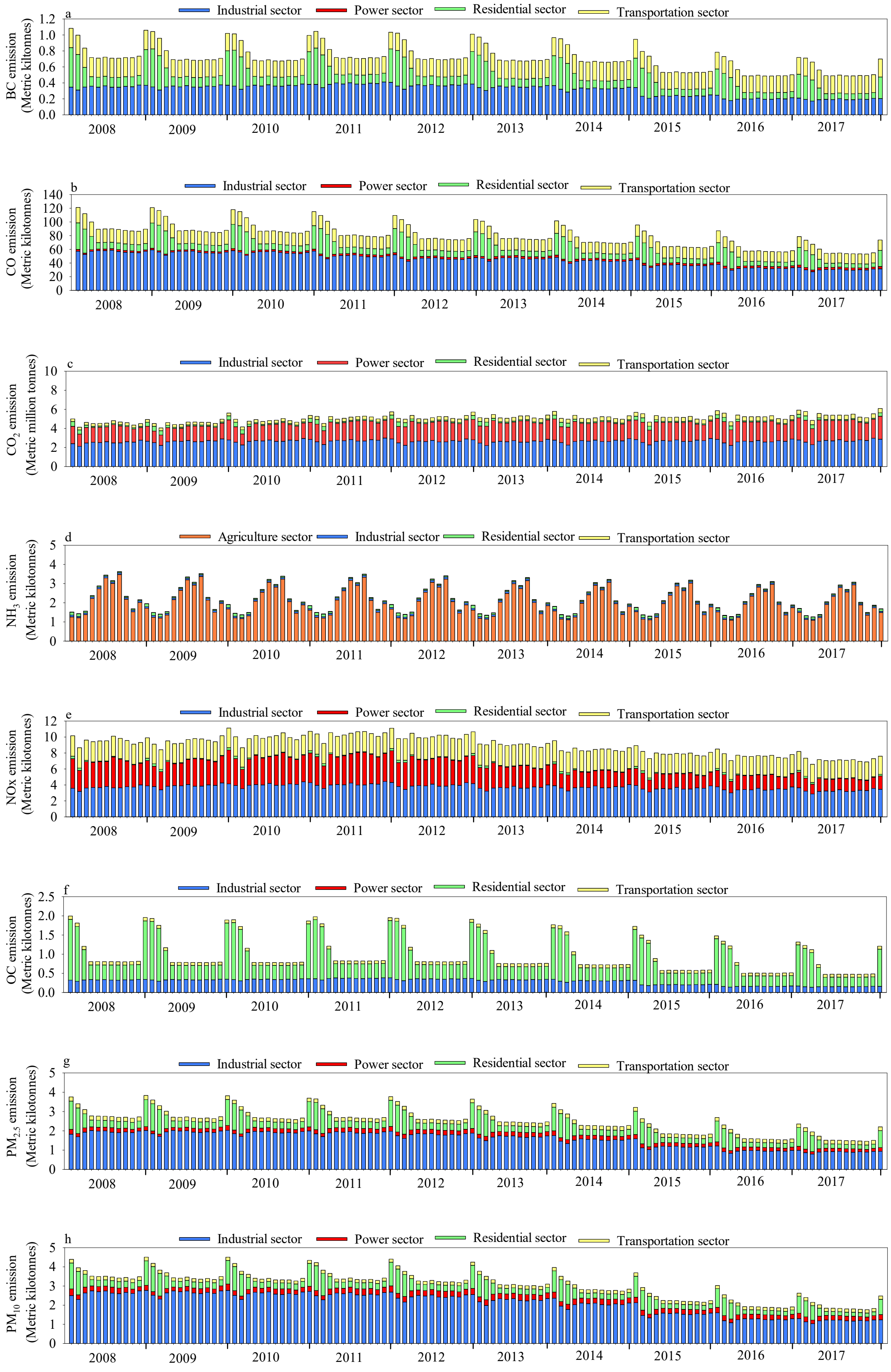
a
Industrial sector
Power sector
Residential sector
Transportation sector
BC emission (Metric kilotonnes)
b
CO emission (Metric kilotonnes)
c
CO_2 emission (Metric million tonnes)
d
Agriculture sector
NH_3 emission (Metric kilotonnes)
e
NOx emission (Metric kilotonnes)
f
OC emission (Metric kilotonnes)
g
$PM_{2.5}$ emission (Metric kilotonnes)
h
PM_{10} emission (Metric kilotonnes)
2008
2009
2010
2011
2012
2013
2014
2015
2016
2017

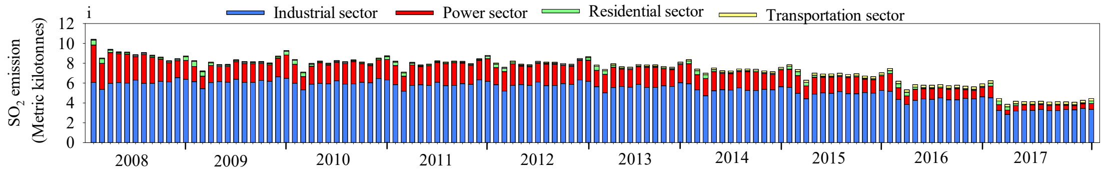

Figure S12. Monthly trends in emissions of air pollutants including BC by sector (a), CO by sector (b), NH_3 by sector (d), NO_X by sector (e), OC by sector (f), $PM_{2.5}$ by sector (g), PM_{10} by sector (h), SO_2 by sector (i), as well as CO_2 by sector (c) in Yantai from the years of 2008-2017.

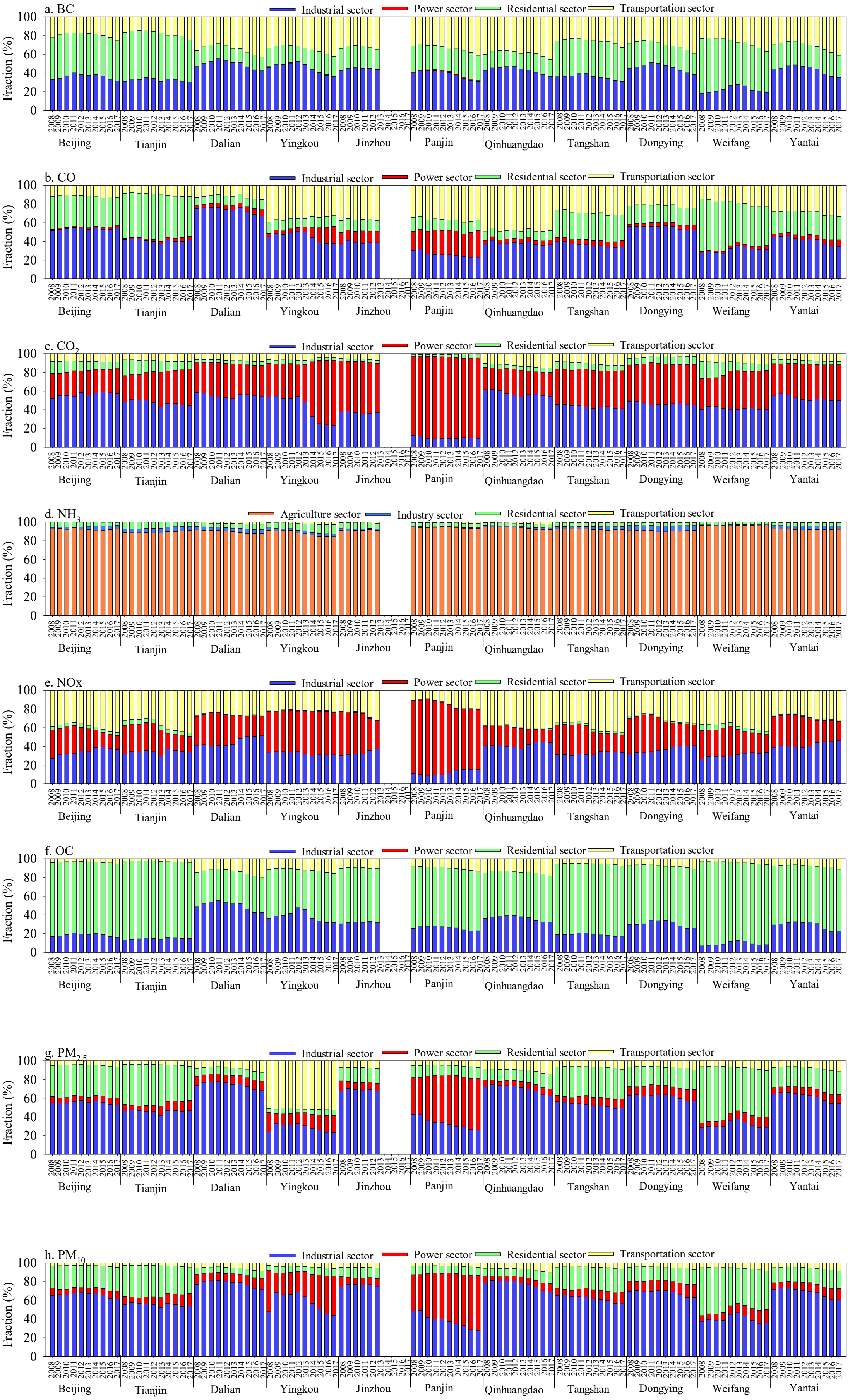
a. BC
b. CO
c. CO_2
d. NH_3
e. NOx
f. OC
g. $PM_{2.5}$
h. PM_{10}
Industrial sector
Power sector
Residential sector
Transportation sector
Agriculture sector
Industry sector
Fraction (%)
0
20
40
60
80
100
2008
2009
2010
2011
2012
2013
2014
2015
2016
2017
Beijing
Tianjin
Dalian
Yingkou
Jinzhou
Panjin
Qinhuangdao
Tangshan
Dongying
Weifang
Yantai

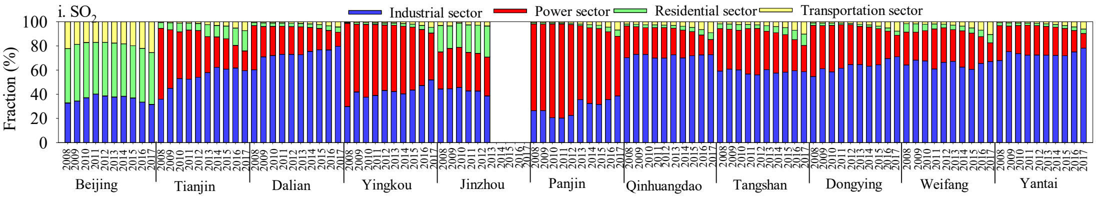

Figure S13. Contribution of the different sectors to the yearly air pollutants including BC (a), CO (b), NH_3 (d), NO_X (e), OC (f), $PM_{2.5}$ (g), PM_{10} (h), SO_2(i), as well as CO_2 (c) in the eleven cities from the years of 2008-2017.

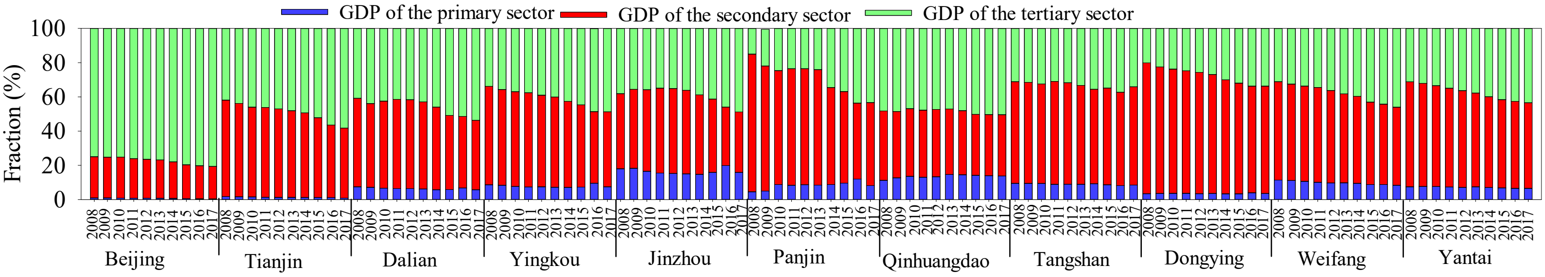

Figure S14. Contributions of the primary sector (agriculture), the secondary sector (industry), and the tertiary sector (excluding agriculture and industry) to GDP from 2008 to 2017 in the 11 cities.

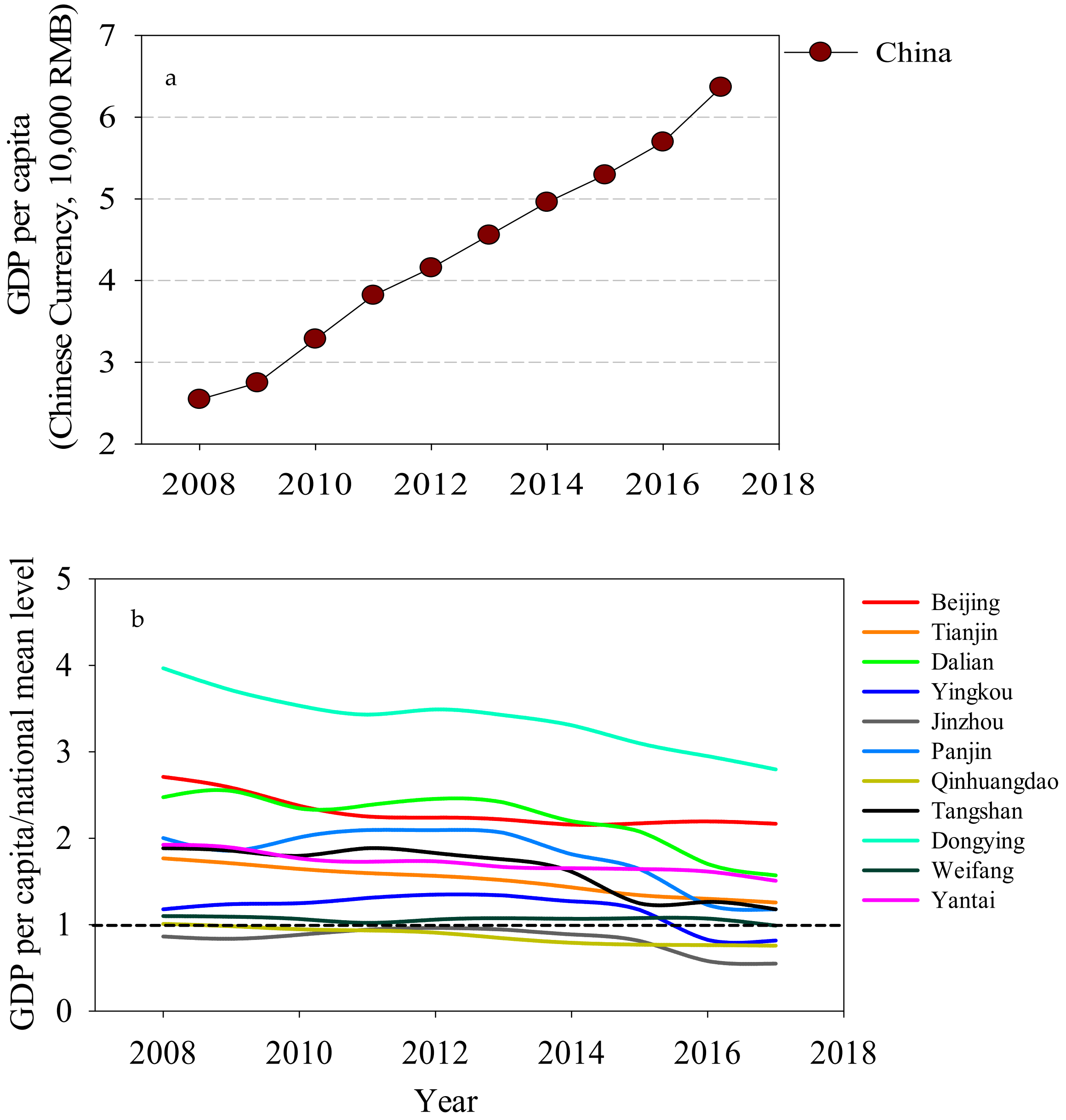

Figure S15. (a) Changes in GDP per capita from 2008 to 2017 in China. (b) Changes in ratio of GDP per capita and national mean level from 2008 to 2017 in 11 cities. Ratio of GDP per capita and national mean level was estimated using GDP per capita for each city divided GDP per capita in China.
